# Enhancing Skin Quality With a Sequential Treatment Using 2 Hyaluronic Acid Dermal Fillers: A Prospective, Multicenter, Interventional Study

**DOI:** 10.1093/asj/sjaf111

**Published:** 2025-06-13

**Authors:** Paula Rosso, Jesús Colina, Carlos Jarne, Eva Garrigós, Bárbara Magalhães, Manuel-Anthony Da Costa, Kevin Poupard

## Abstract

**Background:**

Skin quality affects facial attractiveness, which can significantly impact self-esteem and overall quality of life. The preferred fillers for enhancing skin quality are noncrosslinked or slightly crosslinked hyaluronic acid gels, such as RHA1 and R1, because they diffuse more easily into peripheral tissues.

**Objectives:**

This study was designed to assess the performance and safety of RHA1 treatment for fine lines, such as smiling lines (SLs), including an exploratory arm assessing the performance of subsequent R1 treatments.

**Methods:**

SKINQARE was a prospective, multicenter, low-interventional, open-label study. The primary outcome was Global Aesthetic Improvement Scale (GAIS) score recorded at 2 months postinjection with RHA1. Questionnaires were used to evaluate both patient and investigator satisfaction. Skin quality parameters were measured with standard equipment and the VISIA CANFIELD imaging system. Safety assessment covered adverse events (AEs), injection site pain, and common treatment responses.

**Results:**

The primary endpoint was met, with 92.7% of patients exhibiting GAIS improvement 2 months after receiving RHA1 treatment for SLs. Subsequent treatment with R1 doubled the “very satisfied” rate compared with RHA1 alone at 6 months. Patients also reported prolonged skin firmness, smoothness, and bounce, along with a refreshed feeling. No serious AEs were reported during the study.

**Conclusions:**

RHA1 was effective for skin beautification in all indications, including SLs, and subsequent R1 treatment improved performance. Both treatments were well tolerated. These findings highlight the potential benefits of using a combined treatment approach to improve skin quality.

**Level of Evidence: 3:**

(Therapeutic)

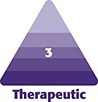

Skin quality plays a crucial role in how age, attractiveness, health, and youth are perceived,^1-4^ with even subtle changes in skin texture or pigmentation significantly affecting facial attractiveness.^5-7^ Consequently, decreased skin quality impacts an individual's emotional health, quality of life, self-perception, and interactions with others.^8^

Skin aging and, therefore, decreased skin quality, results from the gradual loss of hyaluronic acid (HA), subcutaneous adipose tissue, collagen, and elastin fibers.^9^ Among these, HA plays a crucial role in maintaining skin hydration, flexibility, and volume.^10^ Its remarkable capacity to retain water—approximately 1000 times its weight—enables effective hydration of both the epidermis and the dermis.^10,11^

Skin quality can be significantly improved through minimally invasive procedures,^10,12-18^ resulting in sustained enhancements in self-esteem and self-ratings of attractiveness, while decreasing self-perceived age.^19-23^ Previous studies have proposed several attributes that contribute to skin quality, including skin roughness, hydration, elasticity, firmness, and fine lines. However, optimal skin quality is broadly characterized by overall health, youthful appearance, and lack of damage.^1,8^ HA dermal fillers have emerged as a versatile method for addressing multiple facets of skin quality. Specifically, these dermal fillers can be used to address several aspects of skin quality, including hydration, texture, elasticity, plumpness, and collagen stimulation.^24^

In the dermis, HA regulates water balance, osmotic pressure, and ion flow, filters out certain molecules, enhances the extracellular domain of cell surfaces, and stabilizes skin structures through electrostatic interactions.^11^ Injection of the HA dermal filler into the dermis helps to hydrate the skin and supports the formation of a stable extracellular matrix. This matrix is crucial for the proper functioning of fibroblasts in the dermis, ultimately improving skin quality, including elasticity, hydration, and texture.^24^ The hypodermis is also important to consider when addressing skin quality. It lies beneath the dermis and contains adipocytes and large blood vessels, supports keratinocyte and fibroblast proliferation, and incorporates macrophages, fibrous bands, collagen, and elastin that connect the layer to the dermis.^24^ When injected in the hypodermis, HA stimulates procollagen production and induces a stress response in adipose tissue.^25^ This is believed to activate adipose tissue-derived mesenchymal stem cells and their expansion in the scaffold, contributing to prolonged changes in the extracellular matrix and ultimately improving facial appearance.^25^

The properties of HA dermal fillers are influenced by HA concentration, molecular weight, and the degree of crosslinking.^26^ For the correction of superficial of fine lines, the preferred fillers are noncrosslinked or slightly crosslinked and concentrated HA gels, because they diffuse into surrounding tissues, easily adapt to facial dynamism, and are associated with a lower incidence of postprocedural lump formation.^27^ The goal of this beautification treatment is to restore lost volume and smooth out superficial fine lines in targeted facial areas, thereby improving overall skin appearance and texture, and helping the patient look and feel more beautiful. Additionally, noncrosslinked HA can be used to improve skin quality, namely skin texture, plumpness, and firmness, ultimately leading to skin redensification.^8^ Applying a redensification treatment could, thus, lead to healthier, more youthful skin and a refreshed appearance.

RHA1 is a combination of non- and slightly crosslinked high-molecular-weight HA, whereas R1 is a noncrosslinked, high-molecular-weight HA. Both products are indicated for the enhancement of skin quality, the correction of withered skin, and the prevention of superficial fine wrinkles.^28,29^ Real world evidence collected on RHA1 and R1 demonstrates user satisfaction and acceptable safety profiles, with skin quality enhancements among the most commonly performed treatments (data on file). However, further evidence is needed to support the clinical benefit of RHA1 in the perioral region (PO), smiling lines (SLs), and neck lines (NLs), as well as the long-term benefits of using a combination of RHA1 and R1 to enhance skin quality. Thus, a prospective, open-label study, SKINQARE, was designed to assess the performance and safety of skin beautification using RHA1 for fine lines, including an exploratory arm to assess skin redensification by subsequent treatment with R1, with this publication focusing primarily on the use of these 2 dermal fillers on the SLs.

## METHODS

### Study Design and Population

The SKINQARE study (NCT05349799) was a prospective, low-interventional, multicenter, open-labeled study designed to assess the performance and safety of RHA1 for skin beautification of fine lines in the NLs, PO, and SLs, followed by skin redensification by R1. The study was conducted between October 2021 and May 2022 at 4 centers in Spain (Alicante, Barcelona, Bilbao, and Madrid).

Eligible patients were healthy adults, aged 35 to 70 years old, seeking an improvement in their aesthetic facial appearance and beautification of their skin in the NLs, PO, or SLs. The eligible patients had a wrinkle severity ranked as mild to moderate in the NL area (Grade 2 or 3 on the Neck Lines Descriptive Scale) and a wrinkle severity graded as moderate to severe in at least one of the SL area (Grade 3 or Grade 4 on the Smiling Lines Descriptive Scale) or the PO area (Grade 2 or Grade 3 on the Perioral Rythids Severity Rating Scale).^30^ The patients gave their informed consent before participation.

Patients were excluded from the study if they: (1) had already had fillers and/or other skin quality treatment(s) in the 6 months preceding the study; (2) had undergone or planned to undergo peeling treatment or laser/ultrasound-based treatment during the study or had 1 of these treatments in the 6 months preceding the study; (3) had cutaneous disorders, inflammation, or infection (eg, acne, herpes, scars) at or near to the treatment site; (4) had a known hypersensitivity to lidocaine and/or amide local anesthetic agents or HA, or had a history of severe allergy or anaphylactic shock; (5) had an autoimmune or cardiac disease and/or were undergoing treatment for heart disease (beta-blockers); (6) had hepatocellular insufficiency and/or were undergoing treatment for liver disease; (7) were suffering from epilepsy or porphyria; (8) had severe, ongoing, and/or uncontrolled disease that may have posed a health risk to the patient during the study and/or may have had an impact on the study assessments; (9) had received or planned to receive high-dose vitamin E, aspirin, anti-inflammatories, or anticoagulants in the week preceding each injection; (10) were receiving any long-term medical treatment or any treatment that, in the opinion of the clinical investigator, could interfere with test results or put the patient at undue risk; (11) were under guardianship/tutorship; (12) were pregnant or breastfeeding; and (13) were participating in another study or were in an exclusion period for a previous study.

Ethics approval for the sites in Bilbao, Madrid, and Barcelona was obtained from the Comité de Etica de la Investigacion con medicamentos de Euskadi, whereas approval for the site in Alicante was obtained from Comité Etico de investigacion con Medicamentos del Departamento de Salud de Alicante. The investigator of each participating site ensured that the study was conducted in compliance with the Declaration of Helsinki and national regulations applicable to Good Clinical Practice. Informed consent adhered to specific country regulatory requirements. Image rights were obtained via informed consent.

### Study Devices and Treatment Protocol

TEOSYAL RHA 1 (RHA1; Teoxane, Geneva, Switzerland) and TEOSYAL PureSense Redensity 1 (R1; Teoxane) are dermal fillers containing 15 mg/mL HA. RHA1 comprises crosslinked and noncrosslinked HA, whereas R1 comprises only noncrosslinked HA. Both fillers contain lidocaine hydrochloride (0.3% w/w) as a local anesthetic agent to alleviate procedural and postprocedural pain.

**Figure 1.**
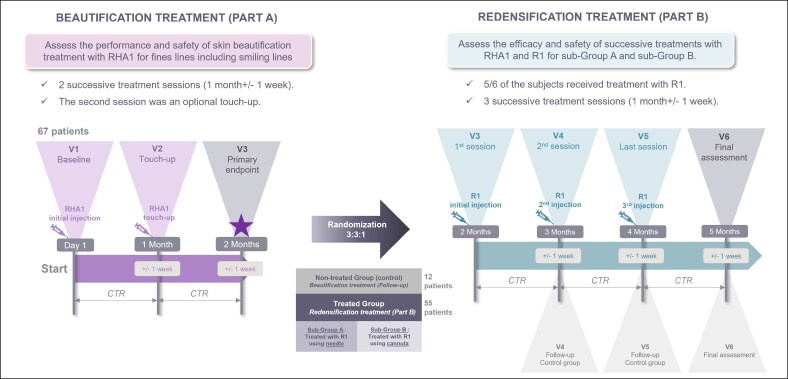
SKINQARE study design.

All included patients were injected with RHA1 at Visit 1 (V1) (Figure 1). Each patient received treatment on the NLs and at least 1 of the other 2 indications (PO or SLs). An optional touch-up with RHA1 could be performed 1 month later (V2) if deemed necessary. Patients were randomized 3:3:1 at V3: 1 out of 6 patients did not receive any treatment and remained in part A of the study (RHA1 only group/control group), whereas 5 out of 6 patients were treated with R1 at 2 months (V3), 3 months (V4), and 4 months (V5) after V1, constituting the RHA1 + R1 group. Before R1 treatment, patients in the RHA1 + R1 group were randomly allocated into 2 subgroups (1:1) to be treated either with a needle (RHA1 + R1n) or with a cannula (RHA1 + R1c). Patients who were not selected to be treated with R1 continued the study without additional treatment (RHA1 only group). Assessments were conducted before injection (V1, if applicable) to 4 months (V5). All patients also attended a final visit (V6, 5 months after V1) to complete final assessments.

The injection technique was chosen at the discretion of the investigator. Injection depth could be either superficial (intradermal) or subcutaneous (in the immediate subdermis) and was dependent on the injection technique and the use of a needle or a cannula. For RHA1, both accessories could be used for all 3 indications, but for R1, the PO region was not to be treated with a cannula. For RHA1 and for R1, the technique used and the total volume per session were collected at each injection visit. A maximal volume of 6 mL could be injected per session, all indications included. Treatment volume per indication was measured to an accuracy of 0.1 mL. Additional anesthetics, beyond the lidocaine included in RHA1 and R1, could be used prior to injection.

### Efficacy and Safety Assessments

#### Aesthetics

Aesthetic improvement was evaluated using the Global Aesthetic Improvement Scale (GAIS) from baseline (V1) to 5 months (V6) by the patients, investigator, and blinded evaluator (BE). The appearance was scored on a scale of 1 to 5, with 1 being “much improved” and 5 being “much worse.”

#### Satisfaction

Satisfaction questionnaires were completed electronically by the patient and investigators and were not anonymous, because they were linked to individual participants for follow-up purposes. Participants had the option to access the survey, distributed by the investigator, via a secure website or through a smartphone application integrated in the Electronic Data Capture system. A blank copy of the satisfaction questionnaires can be accessed in Supplemental Table 1.

Patient satisfaction was assessed at 2 months (V3) and 5 months (V6). For the first question, patients were asked about the main skin benefits of the treatment, to which they could answer “bouncy,” “radiance,” “firmness,” “smoothness,” “hydration”, or “no opinion.” For the second question, patients were asked how their skin feels after treatment and could answer “revitalized,” “refreshed,” “healthy,” “energized,” or “no opinion.” Patients could choose multiple answers for the first 2 questions. The final 2 questions asked the patients to rate their level of satisfaction with the beautification (ie, improvement of your overall skin appearance and feeling in order to look and feel beautiful) and redensification (ie, improvement of the texture, plumpness, and firmness of your skin) of their skin, both of which were answered on a 6-point scale from “very dissatisfied” to “very satisfied.”

Investigator satisfaction was assessed following injection at all visits from baseline to 4 months (V5) for both RHA1 and R1. Injectors were asked whether the product was easy to inject and whether they were satisfied with the product. Only yes/no answers could be given (Supplemental Table 1).

#### Skin Quality

Skin quality was measured by the investigator at 1 month (V2), 2 months (V3), 3 months (V4), and 4 months (V5), prior to any new injection, and at 5 months (V6). Skin hydration was measured with a Corneometer (Courage + Khazaka electronic, Köln, Germany), and skin elasticity was measured with a Cutometer MPA580 (Courage + Khazaka electronic).^25-28^

The VISIA-CR CANFIELD Stereotactic Face and Neck system (Canfield Scientific, Parsippany, NJ) was used to photograph skin roughness/relief (3D photographs, PRIMOS, skin thickness, and wrinkle characteristics (both 2D photographs, VISIA).^29^ The full face and neck were photographed. Of the 27 parameters measured using the CANFIELD system algorithm, the following were kept for analysis after expert's assessment: wrinkle fractional area, mean thickness, *R*_a_ (arithmetic mean of the absolute ordinate value *Z*(*x*)), *R*_v_ (largest pit depth value), wrinkle surface area, wrinkle volume, and percentile wrinkle depth (see Supplemental Table 2).

For skin quality parameters, patients treated for SLs have been compared with the untreated group.

#### Safety

A 100 mm visual analog scale (VAS) was used to measure the patients’ self-assessment of pain immediately after injection and at 5 and 15 min after injection. A score of 0 mm indicated “no pain,” whereas 100 mm indicated “worst pain.”

Immediate common treatment responses (CTRs) were collected by the investigator after each injection. These were defined as expected injection site skin reactions occurring either immediately or in the month following injection of the investigational products. CTRs occurring within the month following each injection were collected by the patient using a diary. CTRs were not classified as adverse events (AEs) unless the severity and/or duration were in excess of that normally observed (as judged by the investigator), or it required medical treatment.

AEs were collected throughout the study by the investigator. Details of the relationship to the device (as judged by the investigator), severity, and seriousness were also recorded.

### Study Endpoints

The primary efficacy endpoint of the study was the proportion of patients deemed “improved” or “much improved” on the GAIS at 2 months (V3) postinjection with RHA1, as assessed by both the investigator and the patient, for all indications combined. The primary endpoint was met if the lower bound of the bilateral 95% CI was ≥70%. Additionally, subpopulation analyses were performed for each individual indication, and this publication presents the results specifically related to SLs.

Secondary efficacy endpoints looked at the performance of RHA1 alone. In this work, these endpoints considered just the SLs. They included the proportion of patients “improved” or “much improved” on the GAIS scale throughout the study (GAIS improvement), as assessed by the investigator and/or the patient, or the BE, and the change in skin quality (roughness, hydration, elasticity, thickness and wrinkle characteristics (Supplemental Table 2) from baseline (V1) to all other visits, as well as patient and investigator satisfaction (Figure 1).

The secondary safety endpoints of the study considered RHA1 alone and included AEs, CTRs reported both immediately and in the month following injection, and injection site pain. Secondary safety endpoints considered all indications to determine the overall safety of the product.

The exploratory efficacy endpoints were the same as the secondary endpoints but considered successive RHA1 and R1 treatments at 3 months (V4), 4 months (V5), and 5 months (V6) compared with RHA1 alone. All exploratory efficacy endpoints are shown for SLs only. The exploratory safety endpoints were the same as the secondary safety endpoints but for successive RHA1 + R1 treatments. Exploratory safety endpoints considered all indications to determine the overall safety of R1.

### Statistical Analysis

All statistical analyses were performed using SAS software version 9.4 (SAS Institute, Cary, NC). Quantitative data were described by their mean (and corresponding 95% CI, if relevant), SD, median, and range (Min, Max). Qualitative data were described by their number (*n*) and percentage (%). Missing data were not replaced.

For the primary endpoint, a unilateral test with bilateral 95% CI was performed with the corresponding *P*-value. For each indication, a paired Student's *t* test or Wilcoxon rank signed test was used to compare results at baseline with those at each follow-up visit, and a *χ*^2^ or Fisher’s exact test was used to compare improvement on the GAIS by methods of injection. For skin quality analysis, analysis of covariance models were used to compare treatment groups. The significance threshold was set at 0.05.

## RESULTS

### Patient Demographics

A total of 67 patients were enrolled in the study across all 4 sites. Of these, 61 (91.0%) were female. Mean age on inclusion was 55.1 ± 7.5 years and ranged from 42 to 69 (Table 1). Most were Caucasian (92.5%) and were Type III (62.7%) or Type II (29.9%) on the Fitzpatrick scale. Less than half of the patients had smoked (43.3%) or consumed alcohol (49.3%) in the past 5 years. No previous medical history associated with HA injections or previous history of facial surgery was reported.

**Table 1.** Patient Demographics

Variable	Participants (*n* = 67)
Age (mean ± SD)	55.1 ± 7.5 years
Gender	
Female	61 (91.0%)
Male	6 (9.0%)
Ethnicity	
Caucasian	62 (92.5%)
Asian	0 (0.0%)
Black or African American	0 (0.0%)
Hispanic	5 (7.5%)
Mixed	0 (0.0%)
Other	0 (0.0%)
Fitzpatrick skin type	
Type I	2 (3.0%)
Type II	20 (29.9%)
Type III	42 (62.7%)
Type IV	3 (4.5%)
Type V	0 (0.0%)
Type VI	0 (0.0%)
Smoking tobacco products	
Currently smoke	17 (25.4%)
Do not currently smoke but have in the past 5 years	12 (17.9%)
Have not smoked in the past 5 years	38 (56.7%)
Consuming alcohol products	
Currently drink	26 (38.8%)
Do not currently drink but have in the past 5 years	7 (10.4%)
Have not drank in the past 5 years	34 (50.7%)

Percentages may not add to 100% because of rounding. *n*, number of patients; SD, standard deviation.

The average patient follow-up time was 4.82 ± 0.26 months after the initial injection. Follow-up visits were performed at 60.6 ± 6.4 days (2 months, *n* = 66), 89.5 ± 7.1 days (3 months, *n* = 66), 118.1 ± 6.7 days (4 months, *n* = 64), and 145.8 ± 7.9 days (5 months, *n* = 63). Ninety-four percent (94.0%) of patients attended all follow-up visits, whereas 4 patients (6.0%) were prematurely lost to follow-up: 1 after V1, 2 after 3 months (V4), and 1 after 4 months (V5).

### RHA1 Treatment, Baseline to 2 Months (V1-V3)

#### Exposure

Overall, 307 RHA1 injections were administered: 158 initial injections at baseline (V1) and 149 touch-up injections at 1 month (V2) (Table 2). At baseline, all patients received NL treatment, with 49 (73.1%) also receiving PO treatment and 42 (62.7%) receiving SL treatment. At 1 month, most patients received a touch-up regardless of the indication.

**Table 2.** RHA1 Treatment Exposure

Treatment exposure (all indications *n* = 67)		
	V1	V2
Total number of injections	158	149

Treatment exposure (smiling lines *n* = 42)		
	V1	V2
Total number of injections	42	39
Use of anaesthetic		
Yes	16 (38.1%)	19 (48.7%)
No	26 (61.9%)	20 (51.3%)
Method of injection		
Needle	9 (21.4%)	19 (48.7%)
Cannula	16 (38.1%)	6 (15.4%)
Both	17 (40.5%)	14 (35.9%)
Total volume injected (mean ± SD)		
Overall	2.5 ± 1.6 mL	
Needle	1.2 ± 0.5 mL	
Canula	2.4 ± 1.7 mL	
Both	3.2 ± 1.5 mL	

Injection depth and technique (smiling lines *n* = 42)			
	Method of injection	V1	V2
Injection depth	Needle (V1 *n* = 9, V2 *n* = 19)	Superficial: 8 (88.9%) Both: 1 (11.1%)	Superficial: 15 (78.9%) Both: 4 (21.1%)
	Cannula (V1 *n* = 16, V2 *n* = 6)	Subcutaneous: 15 (93.8%) Both: 1 (16.3%)	Superficial: 1 (16.7%) Subcutaneous: 2 (33.3%) Both: 3 (50.0%)
	Both (V1 *n* = 17, V2 *n* = 14)	Superficial: 3 (17.6%) Subcutaneous: 1 (5.9%) Both: 13 (76.5%)	Superficial: 2 (14.3%) Both: 12 (85.7%)
Injection technique	Needle (V1 *n* = 9, V2 *n* = 19)	Multiple depots: 8 (77.8%) Serial puncture: 1 (11.1%) Blanching: 1 (11.1%)	Multiple depots: 8 (42.1%) Serial puncture: 2 (10.5%) Blanching: 2 (10.5%) Linear threading: 9 (47.4%)
	Cannula (V1 *n* = 16, V2 *n* = 6)	Fanning: 16 (100.0%) Linear threading: 4 (25.0%)	Fanning: 6 (100.0%) Linear threading: 3 (50.0%)
	Both (V1 *n* = 17, V2 *n* = 14)	Multiple depots: 2 (11.8%) Serial puncture: 3 (17.6%) Blanching: 12 (70.6%) Fanning: 15 (88.2%) Linear threading: 2 (11.8%)	Blanching: 14 (100.0%) Fanning: 13 (92.9%) Linear threading: 2 (14.3%)

Both superficial and subcutaneous techniques. Percentages may not add to 100% because of rounding. For the injection technique, percentages may not add to 100% because of the use of multiple techniques. *n*, number of patients; SD, standard deviation.

Of the 42 initial injections performed in the SLs at baseline, most were executed with a cannula (38.1%) or using both needle and cannula (40.5%) (Table 2). Needle injections were mainly superficial (intradermal; 88.9%), cannula injections were mostly subcutaneous (immediate subdermis; 93.8%), and methods combining both devices were mainly both superficial and subcutaneous. The most common injection techniques were the “multiple depots” technique—either alone or in combination with another technique- using a needle (77.8%), and the “fanning” technique when using a cannula (100.0%) or both needle and cannula (88.8%). Anesthetics were used for 38.1% of initial injections at baseline.

The 39 touch-up injections in the SLs at 1 month (V2) were mostly performed with a needle (48.7%) or with both injection devices (35.9%) (Table 2). Needle injections were mainly superficial (78.9%), cannula injections were both superficial and subcutaneous (50.0%) or subcutaneous (33.0%), and superficially and subcutaneously (85.7%) when both devices were used. The “fanning” technique remained the most used with a cannula and both needle and cannula, whereas the “multiple depots” and “linear threading” techniques were most used either alone or in combination with another technique when using a needle. Anesthetics were used for 48.7% of touch-up injections at 1 month.

The total mean volume of RHA1 administered at baseline and at 1 month for SLs was 2.5 ± 1.6 mL (range, 0.5-6 mL; Table 2). The total mean volume administered was 1.2 ± 0.5 mL when using a needle, 2.4 ± 1.7 mL when using a cannula, and 3.2 ± 1.5 mL when using both.

#### Investigators’ Satisfaction

All the investigators found RHA1 easy to use for SLs at both the first administration and touch-up. They were also satisfied with the product at every injection.

#### Aesthetic Improvement

Of the 158 injections at baseline (V1) for all indications, 153 had an available GAIS score at 2 months (V3). Aesthetic appearance as assessed by the GAIS was deemed “improved” or “much improved” by both the investigator and the patient for 133 of these injections (86.9% [95% CI, 81.6%-92.3%]. As the lower bound of the 95% CI was above the required threshold (70%), the primary endpoint was successfully met.

**Figure 2.**
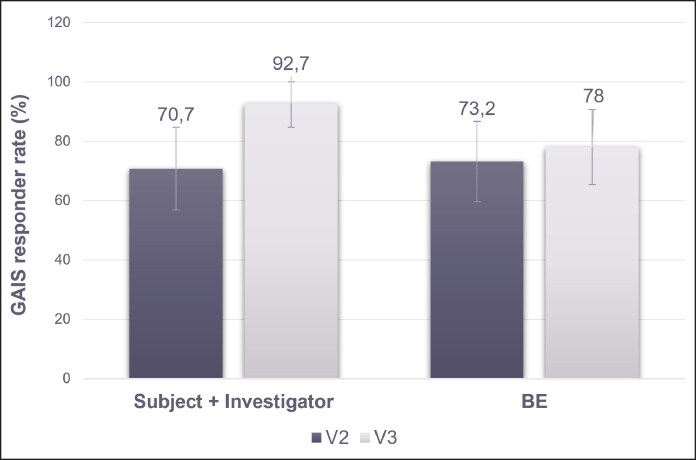
Proportion of patients deemed “improved” or “much improved” on the Global Aesthetic Improvement Scale at 1 month (V2) and 2 months (V3) following RHA1 treatment for SLs, as assessed by the patient plus investigator, and by the BE. BE, blinded evaluator. SLs, smiling lines.

When considering only SLs, 38 out of 41 injections (92.7% [95% CI, 84.7%-100.0%]) achieved GAIS improvement when assessed by both the investigator and the patient at 2 months. The percentage of patients achieving improvement according to both the investigator and the patient increased from 1 to 2 months because of touch-up injections (70.7% vs 92.7%) (Figure 2). GAIS improvement as assessed by the BE also increased slightly from 1 to 2 months (73.2% vs 78.0%). Patients more frequently rated their SL appearance as “much improved” than the investigator or BE.

There was no significant difference in the proportion of patients achieving improvement when assessed by both the investigator and the patient between injection devices.

### R1 Treatment, 2 to 5 Months (V3-V6)

#### Exposure

Fifty-five patients were included in the RHA1 + R1 group (*n* = 28, RHA1 + R1n; *n* = 27, RHA1 + R1c) and 12 in the RHA1-only group. A total of 362 R1 injections were performed (Table 3). Of the injections performed from 2 months (V3) to 4 months (V5), 34 were performed in the SLs at each visit (needle: *n* = 20, cannula: *n* = 14), except for 3 months (V4), which had an additional patient wrongly injected (*n* = 35; needle: *n* = 20, cannula: *n* = 15). Anesthetics were used in between 38.2% and 44.1% of injections, depending on the visit.

**Table 3.** R1 Treatment Exposure

Treatment exposure (all indications *n* = 67)			
	V3	V4	V5
Total number of injections	121	122	119

Treatment exposure (smiling lines *n* = 34)			
	V3	V4	V5
Total number of injections	34	35^a^	34
Use of anaesthetic			
Yes	13 (38.2%)	15 (42.9%)	15 (44.1%)
No	21 (61.8%)	20 (57.1%)	19 (55.9%)
Method of injection			
Needle	20 (58.8%)	20 (57.1%)	20 (58.8%)
Cannula	14 (41.2%)	15 (42.9%)	14 (41.2%)
Total volume injected (mean ± SD)			
Overall	4.7 ± 2.5 mL		
Needle	4.1 ± 2.6 mL		
Canula	5.7 ± 2.2 mL		

Injection depth and technique (smiling lines *n* = 34)				
	Method of injection	V3	V4	V5
Injection depth	Needle (*n* = 20 for V3, V4 and V5)	Superficial: 20 (100.0%)	Superficial: 20 (100.0%)	Superficial: 20 (100.0%)
	Cannula (V3 *n* = 14, V4 *n* = 15, V5 *n* = 14)	Superficial: 10 (71.4%) Subcutaneous: 4 (28.6%)	Superficial: 12 (80.0%) Subcutaneous: 3 (20.0%)	Superficial: 11 (78.6%) Subcutaneous: 3 (21.4%)
Injection technique	Needle (*n*= 20 for V3, V4 and V5)	Multiple depots: 6 (30.0%) Serial puncture: 11 (55.0%) Linear threading: 3 (15.0%)	Multiple depots: 5 (25.0%) Serial puncture: 10 (50.0%) Linear threading: 5 (25.0%)	Multiple depots: 5 (25.0%) Serial puncture: 10 (50.0%) Linear threading: 5 (25.0%)
	Cannula (V3 *n* = 14, V4 *n* = 15, V5 *n* = 14)	Fanning: 13 (92.9%) Linear threading: 4 (28.6%)	Fanning: 11 (73.3%) Linear threading: 7 (46.7%)	Fanning: 10 (71.4%) Linear threading: 7 (50.0%)

Percentages may not add to 100% because of rounding. For the injection technique, percentages may not add to 100% because of the use of multiple techniques. *n*, number of patients; SD, standard deviation.

^a^An additional patient was treated for smiling lines by mistake.

At each visit, all injections made with a needle were superficial (intradermal), and most cannula injections were also superficial (71.4%-80.0%) (Table 3). The most common techniques (alone or in combination) were “serial puncture” when R1 was injected with a needle and the “fanning” technique when using a cannula.

The total mean volume of R1 injected in the SLs across all visits was 4.7 ± 2.5 mL and ranged from 0.6 to 9.0 mL (Table 3). When using a needle, the total volume was 4.1 ± 2.6 mL, whereas the total volume administered using a cannula was 5.7 ± 2.2 mL.

#### Investigators’ Satisfaction

All investigators were satisfied with R1 and considered it easy to inject at V3, V4, and V5 for SLs.

### Comparison between RHA1 only and RHA1 + R1 for smiling lines

#### Aesthetic Improvement

**Figure 3.**
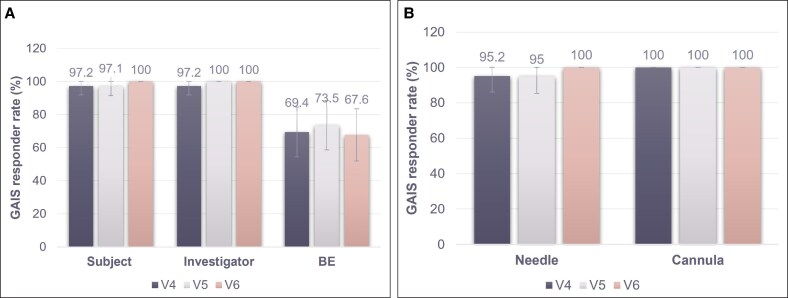
Proportion of patients deemed “improved” or “much improved” on the Global Aesthetic Improvement Scale following RHA1 + R1 treatment. (A) Improvement at 3 months (V4), 4 months (V5), 5 months (V6) for SLs, as assessed by the patient, investigator, and BE. (B) Improvement at 3 months (V4), 4 months (V5), 5 months (V6) for SLs by method of injection, as assessed by the patient plus investigator. BE, blinded evaluator. SLs, smiling lines.

When comparing RHA1 + R1 with RHA1 alone in the SLs, the percentage of patients achieving GAIS improvement according to the patients ranged from 97.1% to 100.0% in the RHA1 + R1 group from 3 months (V4) to 5 months (V6) (Figure 3A), similar to the 100.0% improvement seen with RHA1 alone at each timepoint. Investigator-assessed GAIS results were also comparable, with 97.2% to 100.0% improvement in the RHA1 + R1 group vs 100.0% in the RHA1-alone group. However, BE-assessed GAIS improvement was lower in the RHA1 + R1 group (67.6%-73.5%) compared with RHA1 alone, which ranged from 60.0% to 100.0%, although the latter had a smaller sample size (*n* = 5). Additionally, when both investigator and patient assessments were combined, the RHA1 + R1 group showed at least 95.0% improvement, regardless of whether a needle or cannula was used for R1 injection (Figure 3B).

#### Patient Satisfaction

**Figure 4.**
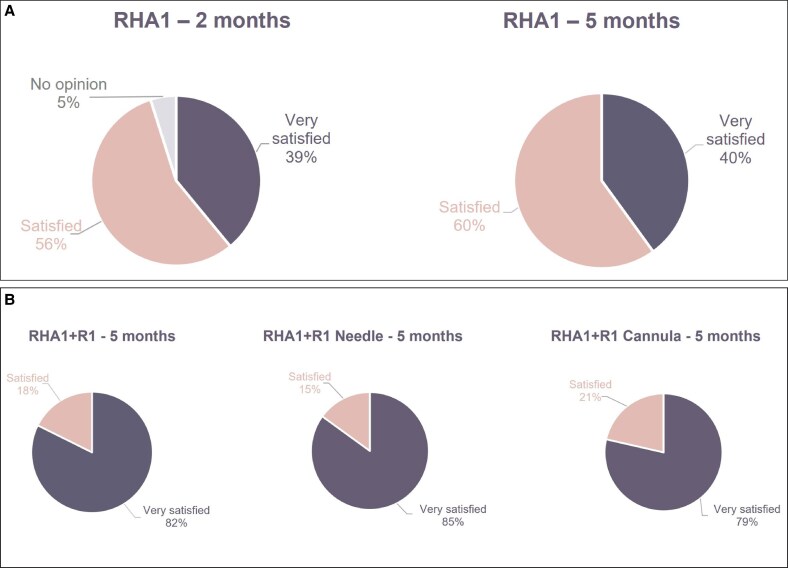
Patient satisfaction following SL treatment. (A) Patient satisfaction at 2 months (V3) and 5 months (V6) following treatment with RHA1. (B) Patient satisfaction at 5 months (V6) after treatment with RHA1 + R1 and by method of injection. SLs, smiling lines.

At 2 months (V3), 16 (39%) patients treated with RHA1 only were “very satisfied,” 23 (56%) were “satisfied,” and 2 (5%) had no opinion (Figure 4A). All patients were still “very satisfied” (40%) or “satisfied” (60%) at 5 months (V6). The proportion of patients in the RHA1 + R1 group who were “very satisfied” at 5 months was 2-fold greater (82%) than the RHA1-only group, and the remainder were “satisfied” (18%) (Figure 4B). There was little difference between needle and cannula at 5 months in the RHA1 + R1 group (Figure 4B). Regardless of the group, no patients were “dissatisfied” or “very dissatisfied” at 2 or 5 months.

**Figure 5.**
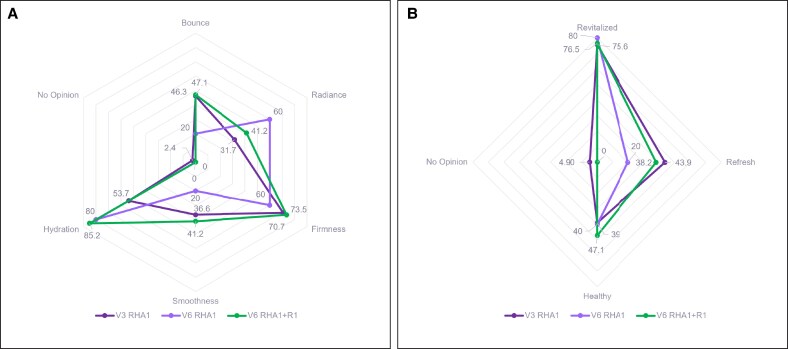
Skin benefits and feelings as assessed by the patient following SLs treatment. (A) Skin benefits at 2 months (V3) and 5 months (V6) following RHA1 treatment, as well as 5 months (V6) following RHA1 + R1 treatment. (B) Skin feelings at 2 months (V3) and 5 months (V6) following RHA1 treatment, as well as 5 months (V6) following RHA1 + R1 treatment. SLs, smiling lines.

The most reported benefit 2 months (V3) following RHA1 injection in SLs was “firmness” (70.7%), whereas the most reported feeling was “revitalized” (75.6%) (Figure 5A, B). At 5 months (V6), the most reported benefit in the RHA1-only group was “hydration” (80.0%), whereas the most reported feeling was “revitalized” (80.0%) (Figure 5A, B). In the RHA1 + R1 group, the most frequently reported benefits were “hydration” (85.2%) and the feeling of being “revitalized” (76.5%) (Figure 5A, B). Additionally, a prolonged effect was observed for “firmness” (73.5%), “smoothness” (41.2%), and the feeling of being “refreshed” (38.2%) after the RHA1 + R1 treatment.

### Skin Quality Assessment

Using Corneometer and Cutometer measures, no clear conclusion could be drawn on skin hydration or skin elasticity between SLs treated with RHA1 alone or RHA1 + R1 and untreated SLs, regardless of the visit.

A total of 15 patients from 1 center were analyzed with the CANFIELD system. Given that the sample size of the RHA1 + R1c subgroup was small, only the RHA1 + R1n subgroup is reported here.

**Figure 6.**
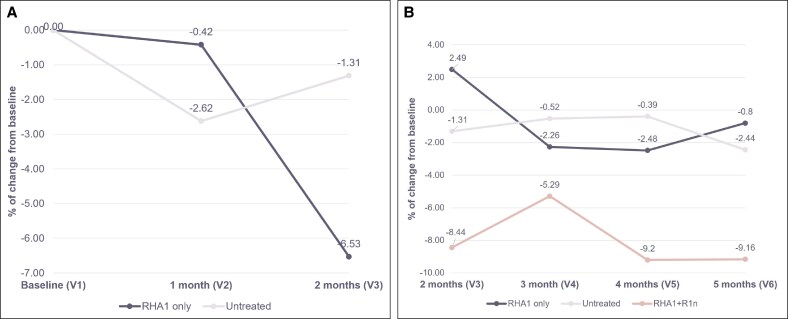
Median change from baseline (%) of mean wrinkle thickness after SLs treatment. (A) Mean wrinkle thickness at baseline (V1), 1 month (V2), and 2 months (V3) for the RHA1-only and untreated groups. (B) Mean wrinkle thickness at 2 months (V3), 3 months (V4), 4 months (V5), and 5 months (V6) for the RHA1-only, untreated, and RHA1 + R1n groups. SLs, smiling lines.

Mean wrinkle thickness decreased following RHA1 treatment compared with baseline by 6.5% at 2 months (V3) (ie, following a touch-up injection of RHA1) and was also lower than the untreated group (Figure 6). In the RHA1-only group, this reduction in mean wrinkle thickness persisted until 4 months (V5). The RHA1 + R1n subgroup prolonged the effect compared with the baseline and the untreated group until 5 months (V6). As observed for the overall RHA1 + R1n, the mean wrinkle thickness of this patient decreased at 3 and 5 months. Although this individual case showed a wrinkle fractional area decrease at 3 and 5 months, overall results were inconclusive because of inconsistent findings from the untreated group (see Supplemental Figure 1).

At 2 months (V3), *R*_a_ (ie, arithmetic mean of the absolute ordinate value *Z*(*x*), Supplemental Figure 2) decreased 10.5% from baseline following RHA1 treatment, compared with 4.8% for the untreated group. This reduction with RHA1 alone persisted up to 3 months (V4), whereas treatment with R1 prolonged this decrease to 5 months (V6).

*R*_v_ (ie, largest pit depth value, Supplemental Figure 3) decreased by 20.0% from baseline at 2 months (V3) following RHA1 treatment, compared with 10.7% in the untreated group, with the reduction lasting until 4 months (V5) for RHA1 alone, whereas R1 prolonged this decrease until 5 months (V6).

Wrinkle surface area decreased by 23.4% at 1 month (V2) and 27.5% at 2 months (V3) following RHA1 treatment, compared with 15.4% and 6.5% in the untreated group (Supplemental Figure 4). The reduction was more pronounced at 3 months (V4) compared with baseline and the untreated group and was prolonged to 5 months (V6) in patients treated with R1.

At 1 month (V2), wrinkle volume had decreased 32.0% from baseline following RHA1 treatment compared with 19.1% in the untreated group (Supplemental Figure 5). By 2 months (V3), the decrease had reached 37.8%, compared with 10.3% in the untreated group. In the RHA1-only group, a decrease compared with baseline and untreated group was prolonged until 5 months (V6), although the reduction at 4 months (V5) was smaller (16.7% lower than baseline compared with at least 35.7% at V3, V4, and V6). The RHA1 + R1n subgroup sustained a decrease of at least 28.1% from baseline between 3 months (V4) and 5 months (V6) and remained lower than the untreated group for this duration.

Percentile wrinkle depth decreased to 17.7% from baseline following RHA1 treatment at 1 month (V2), compared with no change for the untreated group (Supplemental Figure 6). This decrease was more pronounced at 2 months (V3). In the RHA1-only group, the decrease was at least 22.6% below baseline until 5 months (V6). The RHA1 + R1n subgroup exhibited a decrease of 22.4% at 3 months (V4) compared with baseline, which was reduced to 15.5% at 5 months (V6). Both groups also remained lower than the untreated group between 3 (V4) and 5 months (V6).

**Figure 7.**
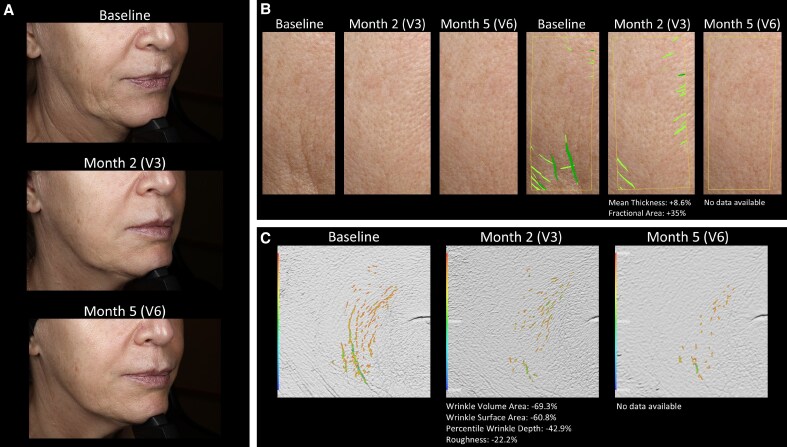
Case 1 of skin quality improvement in a 62-year-old female patient after successive treatment with RHA1 and Redensity 1 (Teoxane) for smiling lines. (A) Patient photographs taken at baseline, 2 months, and 5 months after successive treatment for smiling lines. (B) Skin quality measurements performed with the CANFIELD system at baseline, 2 months (mean thickness: +8.6%; fractional area: +35%), and 5 months after treatment (no data available). (C) Skin texture analysis performed with Primos 3D (Canfield Scientific) at baseline, 2 months (wrinkle volume area: −69.3%; wrinkle surface area: −60.8%; percentile wrinkle depth: −42.9%; roughness: −22.2%), and 5 months after (no data available).

**Figure 8.**
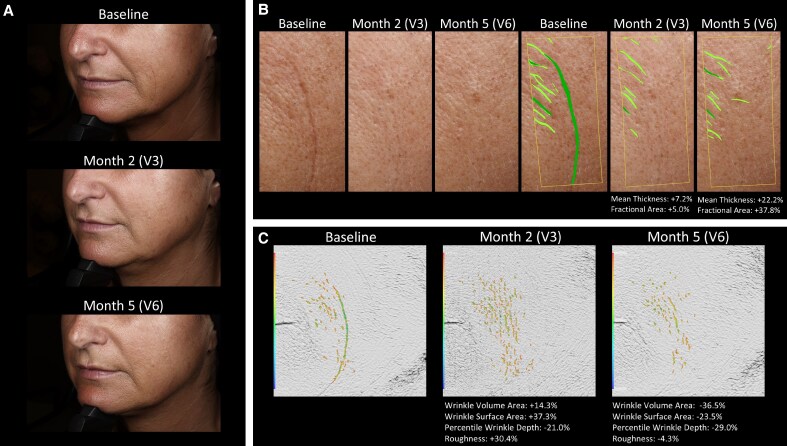
Case 2 of skin quality improvement in a 49-year-old female patient after successive treatment with RHA1 and Redensity 1 (Teoxane) for smiling lines. (A) Patient photographs taken at baseline, 2 months, and 5 months after successive treatment for smiling lines. (B) Skin quality measurements performed with the CANFIELD system at baseline, 2 months (mean thickness: +7.2%; fractional area: +5.0%), and 5 months after treatment (mean thickness: +22.2%; fractional area: +37.8%). (C) Skin texture analysis performed with Primos 3D (Canfield Scientific) at baseline, 2 months (wrinkle volume area: +14.3%; wrinkle surface area: +37.3%; percentile wrinkle depth: −21.0%; roughness: +30.4%), and 5 months after treatment (wrinkle volume area: −36.5%; wrinkle surface area: −23.5%; percentile wrinkle depth: −29.0%; roughness: −4.3%).

Skin quality measurements for 3 patients are illustrated in Figures 7 and 8, and Supplemental Figure 7.

### Safety

#### Injection Site Pain

During treatment with RHA1, the pain felt during injection, as measured by the VAS, was similar for NLs and SLs during V1 initial injections and during touch-up injections at 1 month (V2) (Table 4). Mean pain felt during injection of POs was higher than for other indications at the initial injection and at 1 month (V2). Approximately 75% of patient reported pain under 30.0 mm in the NLs and SLs and under 55.0 mm in the POs; however, in some patients, scores reached as high as 90.0 mm.

**Table 4.** Injection Site Pain Following Treatment

Visit			NLs	POs	SLs
V1 (RHA1)	VAS during injection	*n*	67	49	42
		Mean ± SD	20.9 ± 15.8	40.9 ± 22.9	23.3 ± 17.8
	VAS at end of visit	*n*	67	49	42
		Mean ± SD	0.0 ± 0.0	0.4 ± 2.0	0.0 ± 0.0
V2 (RHA1)	VAS during injection	*n*	65	45	39
		Mean ± SD	20.0 ± 17.5	31.4 ± 24.0	18.5 ± 16.4
	VAS at end of visit	*n*	65	45	39
		Mean ± SD	0.0 ± 0.0	0.4 ± 3.0	0.0 ± 0.0
V3 (R1)	VAS during injection	*n*	53	34	34
		Mean ± SD	20.5 ± 17.0	34.1 ± 20.8	22.9 ± 17.7
	VAS at end of visit	*n*	53	34	34
		Mean ± SD	0.4 ± 1.9	0.0 ± 0.0	0.3 ± 1.7
V4 (R1)	VAS during injection	*n*	53	34	35
		Mean ± SD	23.2 ± 22.9	34.8 ± 24.2	25.4 ± 18.2
	VAS at end of visit	*n*	53	34	35
		Mean ± SD	0.0 ± 0.0	0.0 ± 0.0	0.0 ± 0.0
V5 (R1)	VAS during injection	*n*	52	33	34
		Mean ± SD	25.4 ± 18.2	34.8 ± 24.3	24.8 ± 16.6
	VAS at end of visit	*n*	52	33	34
		Mean ± SD	0.0 ± 0.0	0.0 ± 0.0	0.0 ± 0.0

*n*, number of patients; NL, neck line; PO, perioral region; SD, standard deviation; SL, smiling line; VAS, visual analog scale.

For R1, the pain score during injection remained consistent across the 3 visits for all indications (Table 4). Mean pain scores were again similar for SLs and NLs, with higher pain scores reported in the POs. Over 75% of patients reported pain under 50.0 mm, but some reported pain as high as 100.0 mm.

Regardless of the indication, pain drastically decreased following injection for both products, with mean pain scores approaching zero at 15 min postinjection. Similar pain scores were observed between RHA1 + R1n and RHA1 + R1c subgroups, indicating that the use of a needle or a cannula did not impact perceived pain.

#### Common Treatment Responses

Most CTRs reported by the investigator immediately following RHA1 or R1 injection were mild or moderate in intensity, other than 4 cases of pain following RHA1 treatment and 7 cases of pain following R1 treatment, which were deemed severe. Redness was the most frequently reported CTR for both products, but bleeding, swelling, and pain were also often reported (Table 5). Diaries kept by the patients frequently reported redness, bruising, and lumps/bumps following RHA1 treatment. These were mostly deemed mild to moderate and disappeared within 6 days following the injection. Following R1 treatment, patients often reported lumps/bumps, redness, and swelling, all of which resolved within 2 days. Two CTRs were severe: 1 case of lumps/bumps and 1 case of bruising (both cannula injections). Both severe CTRs resolved within 1 day.

**Table 5.** Common Treatment Responsess Reported by the Investigator Following Treatment

Visit	CTR	Number (%)
V1 (RHA1) (*n* = 158)	Mild redness	137 (86.7)
	Mild swelling	64 (40.5)
	Mild bleeding	30 (19.0)
	Mild bruising	29 (18.4)
	Mild pain	29 (18.4)
	Moderate redness	3 (1.9)
	Moderate swelling	2 (1.3)
	Moderate bleeding	1 (0.6)
	Moderate bruising	3 (1.9)
	Moderate pain	3 (1.9)
	Severe pain	3 (1.9)
V2 (RHA1) (*n* = 149)	Mild redness	90 (60.4)
	Mild swelling	20 (13.4)
	Mild bleeding	39 (26.2)
	Mild pain	23 (15.4)
	Mild lumps/bumps	25 (16.8)
	Moderate redness	24 (16.1)
	Moderate swelling	21 (14.1)
	Moderate bleeding	1 (0.7)
	Moderate pain	7 (4.7)
	Moderate lumps/bumps	4 (2.7)
	Severe pain	1 (0.7)
V3 (R1) (*n* = 121)	Mild redness	76 (62.8)
	Mild swelling	31 (25.6)
	Mild bleeding	36 (29.8)
	Mild pain	15 (12.4)
	Mild lumps/bumps	30 (24.8)
	Moderate redness	2 (1.7)
	Moderate swelling	3 (2.5)
	Moderate bleeding	2 (1.7)
	Moderate pain	11 (9.1)
	Moderate lumps/bumps	2 (1.7)
	Severe pain	1 (0.8)
V4 (R1) (*n* = 122)	Mild redness	76 (62.3)
	Mild swelling	33 (27.0)
	Mild bleeding	34 (27.9)
	Mild bruising	23 (18.9)
	Mild pain	21 (17.2)
	Mild lumps/bumps	24 (19.7)
	Moderate redness	2 (1.6)
	Moderate bleeding	6 (4.9)
	Moderate bruising	1 (0.8)
	Moderate pain	11 (9.0)
	Severe pain	3 (2.5)
V5 (R1) (*n* = 119)	Mild redness	75 (63.0)
	Mild swelling	32 (26.9)
	Mild bleeding	36 (30.3)
	Mild pain	20 (16.8)
	Mild lumps/bumps	24 (20.2)
	Moderate redness	3 (2.5)
	Moderate bleeding	6 (5.0)
	Moderate pain	8 (6.7)
	Severe pain	3 (2.5)

CTR, common treatment response; *n*, number of patients.

#### Adverse Events

Across all indications, 37 AEs were reported in 25 patients (37.3%). Among these AEs, 12 were related to the study device or procedure, from which 11 were CTRs that escalated to AEs (firmness: *n* = 6; lumps/bumps: *n* = 4; bruising: *n* = 1) and 1 was a case of herpes simplex. All these AEs were of mild or moderate intensity, except the case of bruising and case of lumps/bumps reported above. Three AEs were serious (a case of breast cancer, COVID-19-related pneumonia requiring hospitalization, and uterine leiomyoma), but none of these were related to the study device or procedure.

## DISCUSSION

Skin quality plays an important role in perceived facial attractiveness, as well as an individual's self-perception and quality of life,^1-8^ with minimally invasive procedures shown to improve skin quality and thus self-esteem and perceived attractiveness.^10,12-23^ The SKINQARE study used a complex design to show proof-of-concept of the use of RHA1 in skin beautification, followed by R1 for skin redensification. The primary efficacy endpoint of the study was reached, with over 70% of patients achieving improvement on the GAIS at 2 months’ postinjection with RHA1 for all indications, as assessed by both the investigator and the patient. Considering SLs treatment, GAIS improvement approached 100% and all patients were “satisfied” or “very satisfied,” regardless of whether they were treated with RHA1 alone or RHA1 + R1. The benefit of adding R1 to the treatment plan was demonstrated by the percentage of patients who were “very satisfied” with treatment, which was 2-fold higher than RHA1 alone. Patients also reported that their skin felt more refreshed and reported enhanced firmness, smoothness, and bounce with the combination treatment. Although sample size was small, data obtained from the CANFIELD system revealed that RHA1 treatment reduced skin roughness, as well as wrinkle thickness, depth, surface area and volume, with effects lasting 2 to 3 months’ postinjection. Notably, the addition of R1 to the treatment regimen stabilized RHA1's effects, particularly in reducing wrinkle thickness and skin roughness. R1 appeared to prolong some of RHA1's effects for up to 5 months. These findings suggest that the addition of R1 enhances patient satisfaction and prolongs effects on skin quality.

The standard equipment used in this study (Corneometer and Cutometer) did not show any effect of treatment. However, this equipment seems more complex to be used in multicenter clinical studies. These devices are prone to high variability measures because of the use of probes, making the pressure on the skin difficult to standardize.^31^ The CANFIELD system appeared to provide more reliable results, but additional research is necessary to confirm the robustness of CANFIELD measurements and to determine its suitability as an alternative to the standard equipment for measuring skin quality.

GAIS improvement, as assessed by the BE, was generally lower compared with the evaluations provided by the investigators and patients. An exception was observed for the RHA1-only group at 4 and 5 months, in which GAIS improvement reached 100%. This result can be attributed to the small sample size evaluated at these time points (*n* = 5), which may have amplified the perceived effectiveness because of the limited data available.

The BE assessed aesthetic improvement using grading scales during each follow-up visit (from 1 to 5 months), relying solely on photographs. However, feedback from investigators reported discrepancies between the live evaluation of treatment effectiveness and what could be observed in the photographs. These findings were investigated further, and it was confirmed that assessing aesthetic improvement through these photographs was challenging, probably compromising the BE's assessment. Photographs can sometimes fail to capture subtle improvements in skin quality that are more noticeable in person, with factors like lighting, angles, and image resolution potentially leading to more conservative evaluations. As a result, the primary objective was changed from a BE assessment to a combined assessment by the principal investigator (PI) and the patient. Although live assessments were not used in this trial, they are generally preferred in clinical studies, particularly in the field of aesthetics, because of their ability to capture subtle changes and nuances in skin quality that might not be visible in photographs. This should be considered when designing future studies to enhance the reliability of skin quality evaluations.

Both products were well tolerated in all indications with no serious study-related AEs. Most CTRs were mild or moderate and resolved in several days. CTRs were more common but are expected following filler injection.^32^ The pain experienced during injection was mild to moderate for most patients and almost completely resolved in the following 15 min. Efficacy and safety findings for R1 were similar regardless of whether a needle or cannula was used for injection. These findings support the use of RHA1 for skin beautification and suggest that including R1 in the treatment plan can have additional benefits for patients. Overall, both products were well tolerated across all indications, with consistent efficacy and safety outcomes for R1, irrespective of the injection method used.

Although the authors of this study provide valuable insights, it is important to acknowledge some limitations that may impact the interpretation of their findings. For example, this study was limited by the small sample size. Additionally, the 5-month follow-up period may be considered too short to fully assess the long-term efficacy and safety of the sequential treatment with RHA1 and R1 for improving skin quality. A larger study with an extended follow-up period would be valuable to observe the effect of R1 beyond 1 month without additional injections, determine the performance duration of both fillers, and evaluate whether R1 prolongs the effect compared with RHA1 alone. Such a study would also help in assessing potential long-term side effects more comprehensively. With regard to patient demographics, this study was conducted in Spain, where the majority of the population is Caucasian and, therefore, predominantly falls within Fitzpatrick Skin Types II and III. This lack of ethnic and skin type diversity limits the generalizability of the results across diverse populations. Nonetheless, the SKINQARE study successfully demonstrated proof-of-concept for skin beautification with RHA1, followed by skin redensification with R1.

Because treatment in the SLs was optional, the untreated group could be used as a comparison when assessing skin quality using objective measures. This comparison was crucial because we observed fluctuations in measurements in the untreated group. Indeed, for wrinkle fractional area, the results were inconclusive because of a decrease in the untreated group. However, skin quality is a complex concept, with many contributing parameters.^1,8^ Despite these parameters often depending on one another, they are frequently measured in isolation.^8^ The SKINQARE study included a number of skin quality assessments to attempt to capture the complexity of skin quality evaluation. Interestingly, despite the limited sample size, several interesting correlations were demonstrated that should be further investigated in larger studies.

Understanding skin quality involves recognizing that it can be impacted by deeper skin layers such as the hypodermis.^1^ In the SKINQARE study, although all R1 injections with a needle were superficial, those performed with a cannula were occasionally subcutaneous. Despite this, both methods yielded high GAIS scores and satisfaction levels. This implies that favorable outcomes can be achieved using R1 in both the superficial and the subcutaneous planes.

## CONCLUSIONS

The sequential use of 2 HA dermal fillers with distinct compositions offers a holistic approach to improve skin quality, specifically for the treatment of fine lines, including SLs. RHA1 is effective for skin beautification in the SLs, whereas R1 can be administered with a needle or a cannula can be used as an additional redensification step to increase patient satisfaction and potentially prolong the effects of RHA1. Additionally, both devices are well tolerated. However, further studies are needed to measure additional skin quality outcomes on a larger sample size and to define tools for objectively measuring the satisfaction of treated patients.

## Supplemental Material

This article contains supplemental material located online at https://doi.org/10.1093/asj/sjaf111.

## Supplementary Material

sjaf111_Supplementary_Data
