# Supplementary material for: Enhancing Skin Quality With a Sequential Treatment Using 2 Hyaluronic Acid Dermal Fillers: A Prospective, Multicenter, Interventional Study
Source: Aesthet Surg J. 2025 Jun 13;45(10):1051–64. doi: 10.1093/asj/sjaf111 (PMC12448579; doi:10.1093/asj/sjaf111)
Supplement: sjaf111_Supplementary_Data [file sjaf111_supplementary_data.zip › Supplemental Table 1.doc]

Supplemental Table 1. Subject and investigator satisfaction questionnaires. 
Subject satisfaction	
Type	Propositions	Frequency	

Rate the degree of aesthetic improvement for this indication by using the following scale:	·	Much improved
·	Improved
·	No change
·	Worse
·	Much worse
·	Not applicable	Before a new injection, from V2 to V6	
What are the main skin benefits from the treatment?	·	Bouncy
·	Radiance
·	Firmness
·	Smoothness
·	Hydration
·	No opinion	Before a new injection at V3 and at the last visit V6	
How does your skin feel this treatment? My skin feels…	·	Revitalized
·	Refresh
·	Healthy
·	Energized
·	No opinion	Before a new injection at V3 and at the last visit V6	
Beautification is defined by an improvement of your overall skin appearance & feeling in order to look and feel beautiful.

You've been treated with RHA 1, please rate your level of satisfaction regarding the beautification of your skin.	·	Very satisfied
·	Satisfied
·	Neither satisfied nor dissatisfied
·	Dissatisfied
·	Very Dissatisfied
·	No opinion	Before a new injection
at V3 for all patients and at the last visit V6 for patient treated only with RHA1	
Redensification is defined by an improvement of the texture, plumpness and firmness of your skin.

You've been treated with RHA1 and then with Redensity 1, please rate your level of satisfaction regarding the redensification of your skin.	·	Very satisfied
·	Satisfied
·	Neither satisfied nor dissatisfied
·	Dissatisfied
·	Very Dissatisfied
·	No opinion	At V6 only for patient treated with Redensity 1	
Investigator satisfaction	
Type	Propositions	Frequency	
Was TEOSYAL RHA 1/R easy to inject?	Yes/No
Comment	N/A	
Are you satisfied with this product?	Yes/No
Comment	N/A	
Add here any additional comments regarding TEOSYAL RHA 1/R1	Comment	N/A	
If the product was not easy to inject and/or you are not satisfied with product, is this due to a device deficiency?	Yes/No	N/A	
