# Supplementary material for: Enhancing Skin Quality With a Sequential Treatment Using 2 Hyaluronic Acid Dermal Fillers: A Prospective, Multicenter, Interventional Study
Source: Aesthet Surg J. 2025 Jun 13;45(10):1051–64. doi: 10.1093/asj/sjaf111 (PMC12448579; doi:10.1093/asj/sjaf111)
Supplement: sjaf111_Supplementary_Data [file sjaf111_supplementary_data.zip › Supplemental Table 2.docx]

**Supplemental Table 2:** Summary of skin quality parameters.

| **Parameter** | **Description** | **Reason for inclusion** |
| --- | --- | --- |
| **Mean Thickness** | Average thickness of each detected wrinkle | Good 2D measurements to show whether wrinkle widths are reducing and therefore eliminating bigger wrinkles |
| **Wrinkle Fractional Area** | Area of wrinkles detected  divided by total area of interest (AOI) | Good 2D measurement to quantify whether wrinkles are reducing |
| **Ra** | Arithmetic mean of the absolute ordinate value Z(x) | Good measure to quantify general roughness of the skin |
| **Rv** | Largest pit depth value | A measure of wrinkle depth |
| **Wrinkle Surface Area** | Total area of the wrinkle detection along the skin surface | Good measure to quantify surface area of wrinkles and their change over time |
| **Wrinkle Volume** | Total volume formed between the skin surface of the wrinkle detection and the smooth interpolated surface | Good measure to quantify volumes of wrinkles and their change over time |
| **Percentile Wrinkle Depth** | 99^th^ percentile distance between the skin surface of the wrinkle detection and the smooth interpolated surface | Eliminates any noise errors at the higher end of wrinkle depth and can quantify the change of wrinkle depth over time |
