# Supplementary figures and images for: Enhancing Skin Quality With a Sequential Treatment Using 2 Hyaluronic Acid Dermal Fillers: A Prospective, Multicenter, Interventional Study
Source: Aesthet Surg J. 2025 Jun 13;45(10):1051–64. doi: 10.1093/asj/sjaf111 (PMC12448579; doi:10.1093/asj/sjaf111)

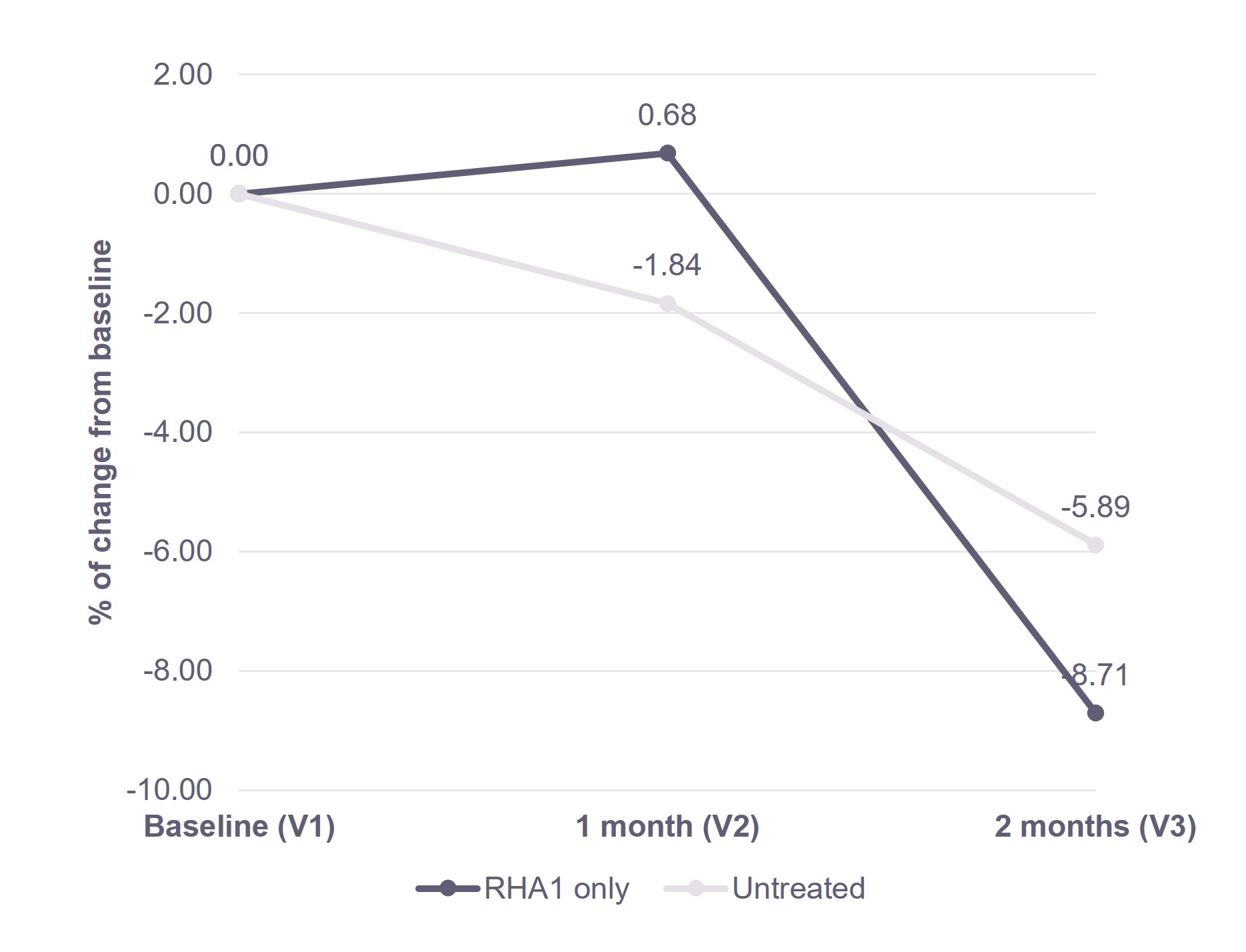

Supplement: sjaf111_Supplementary_Data [file sjaf111_supplementary_data.zip › Supplemental Figure 1A.jpg]

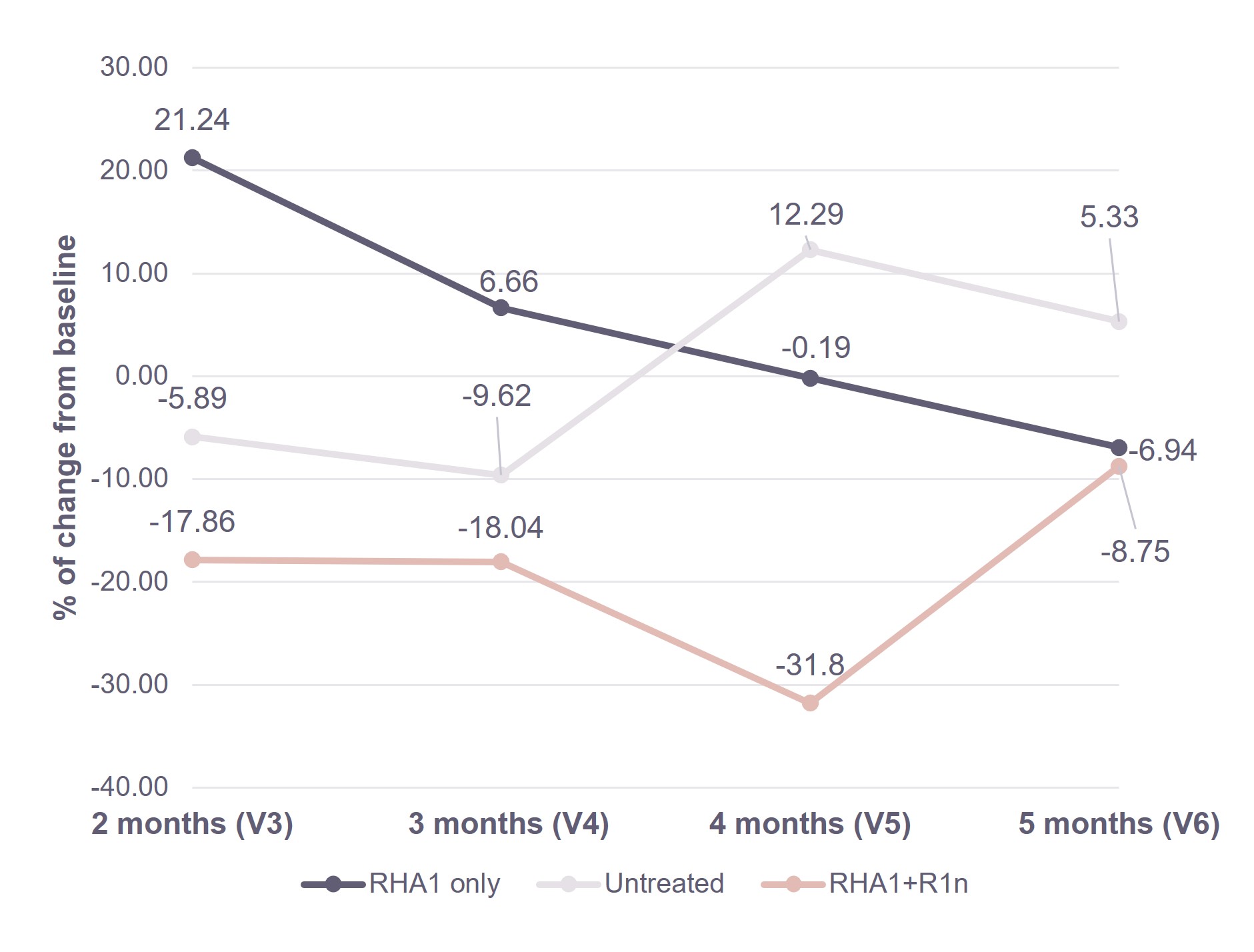

Supplement: sjaf111_Supplementary_Data [file sjaf111_supplementary_data.zip › Supplemental Figure 1B.jpg]

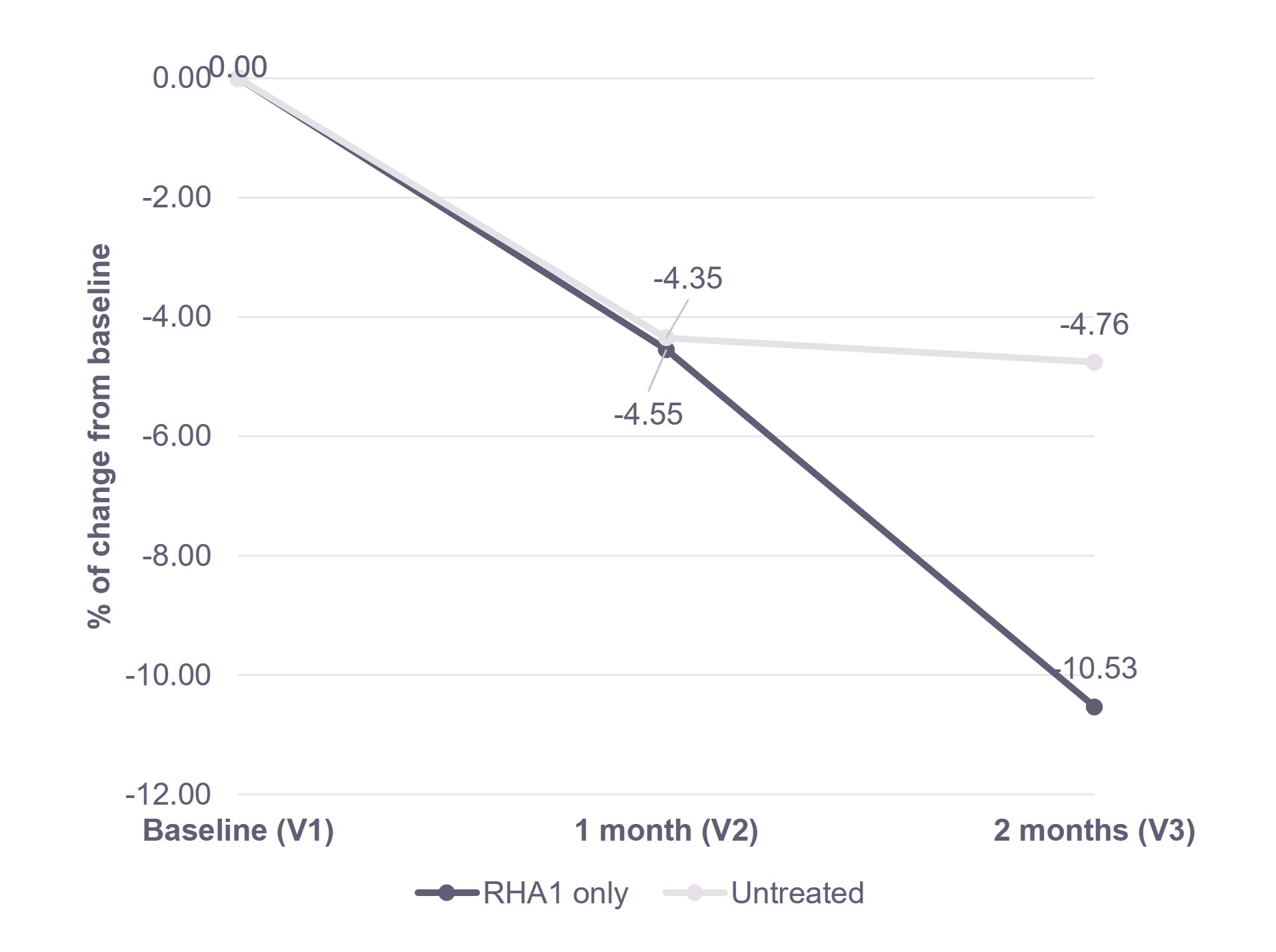

Supplement: sjaf111_Supplementary_Data [file sjaf111_supplementary_data.zip › Supplemental Figure 2A.jpg]

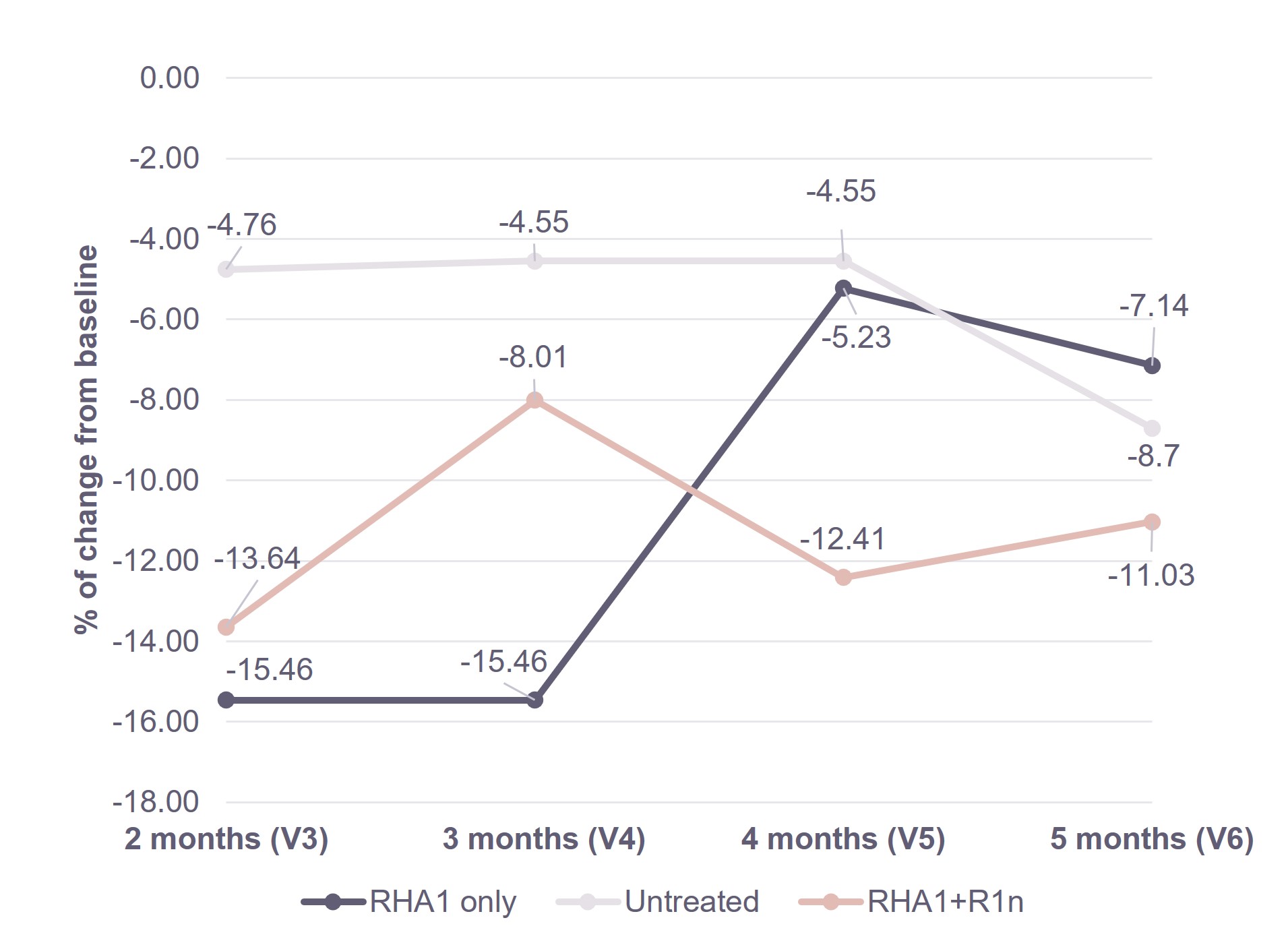

Supplement: sjaf111_Supplementary_Data [file sjaf111_supplementary_data.zip › Supplemental Figure 2B.jpg]

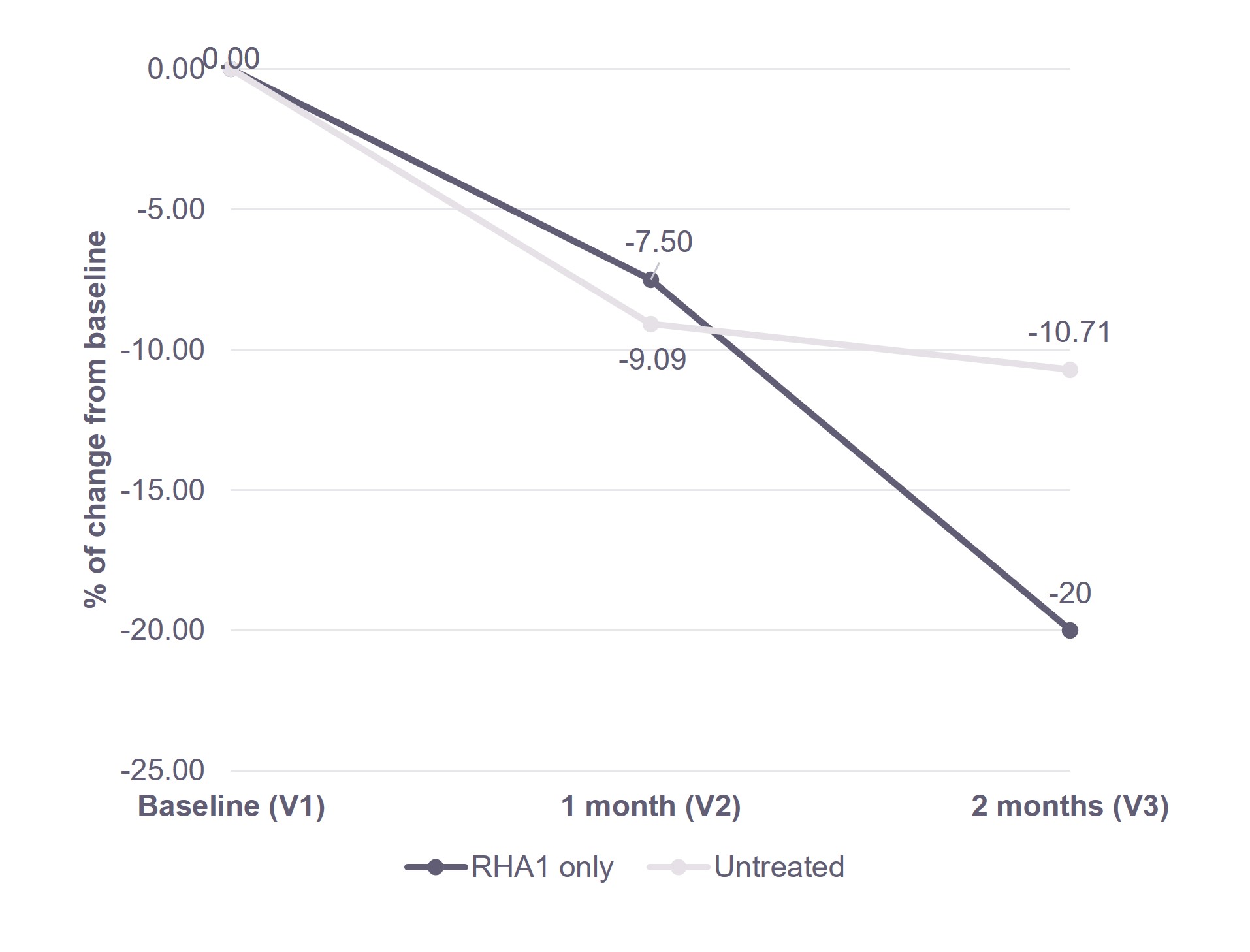

Supplement: sjaf111_Supplementary_Data [file sjaf111_supplementary_data.zip › Supplemental Figure 3A.jpg]

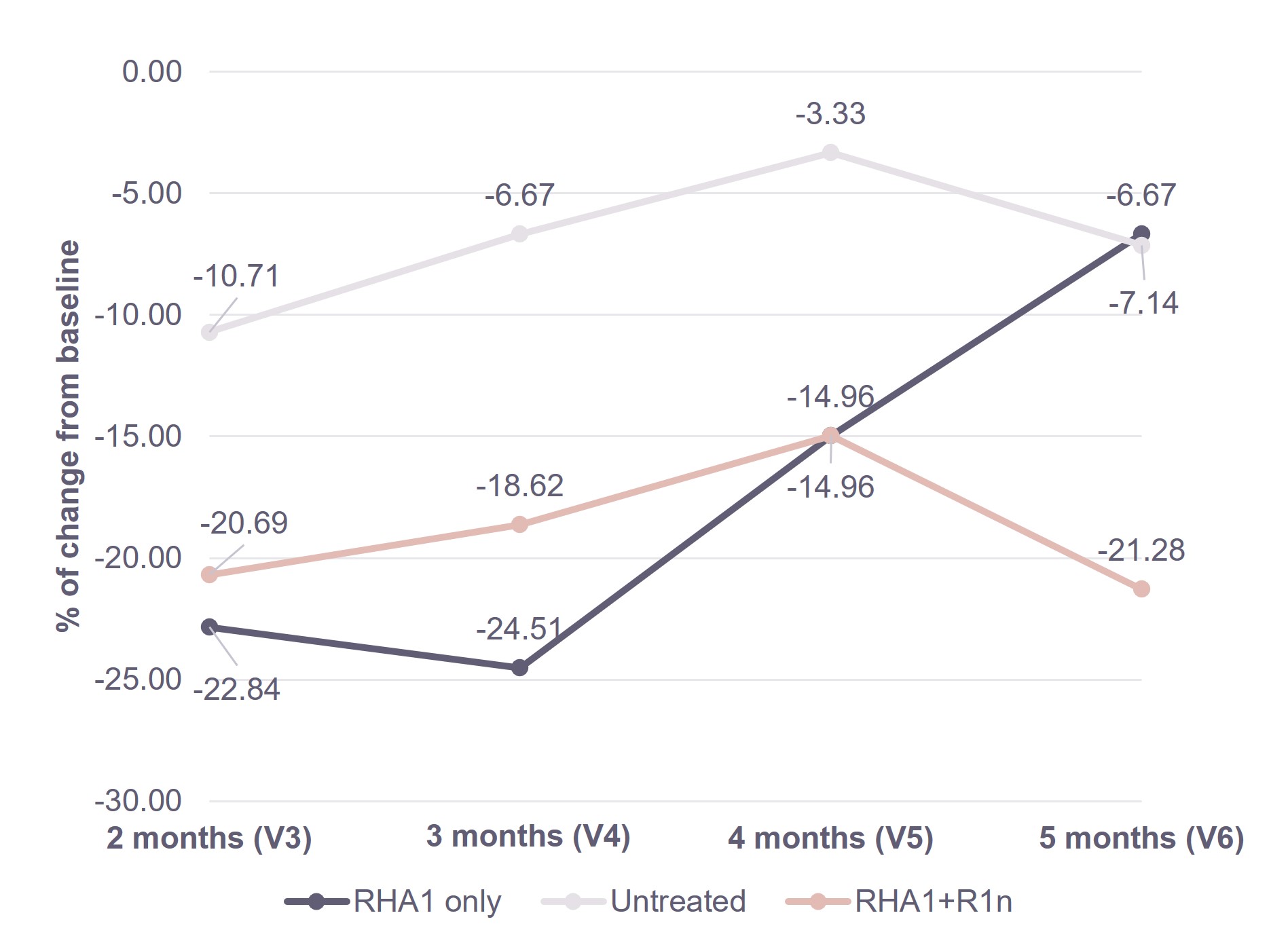

Supplement: sjaf111_Supplementary_Data [file sjaf111_supplementary_data.zip › Supplemental Figure 3B.jpg]

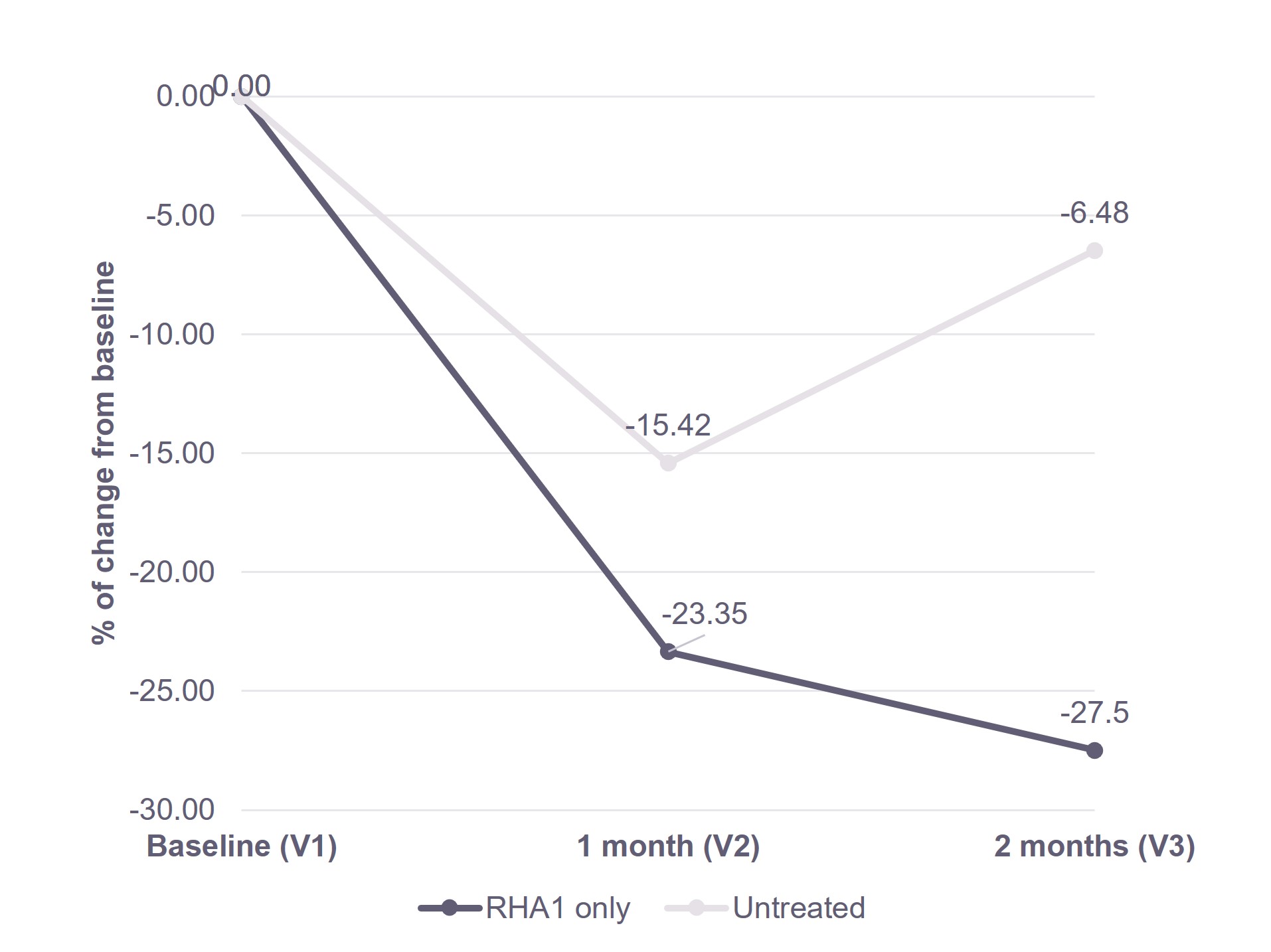

Supplement: sjaf111_Supplementary_Data [file sjaf111_supplementary_data.zip › Supplemental Figure 4A.jpg]

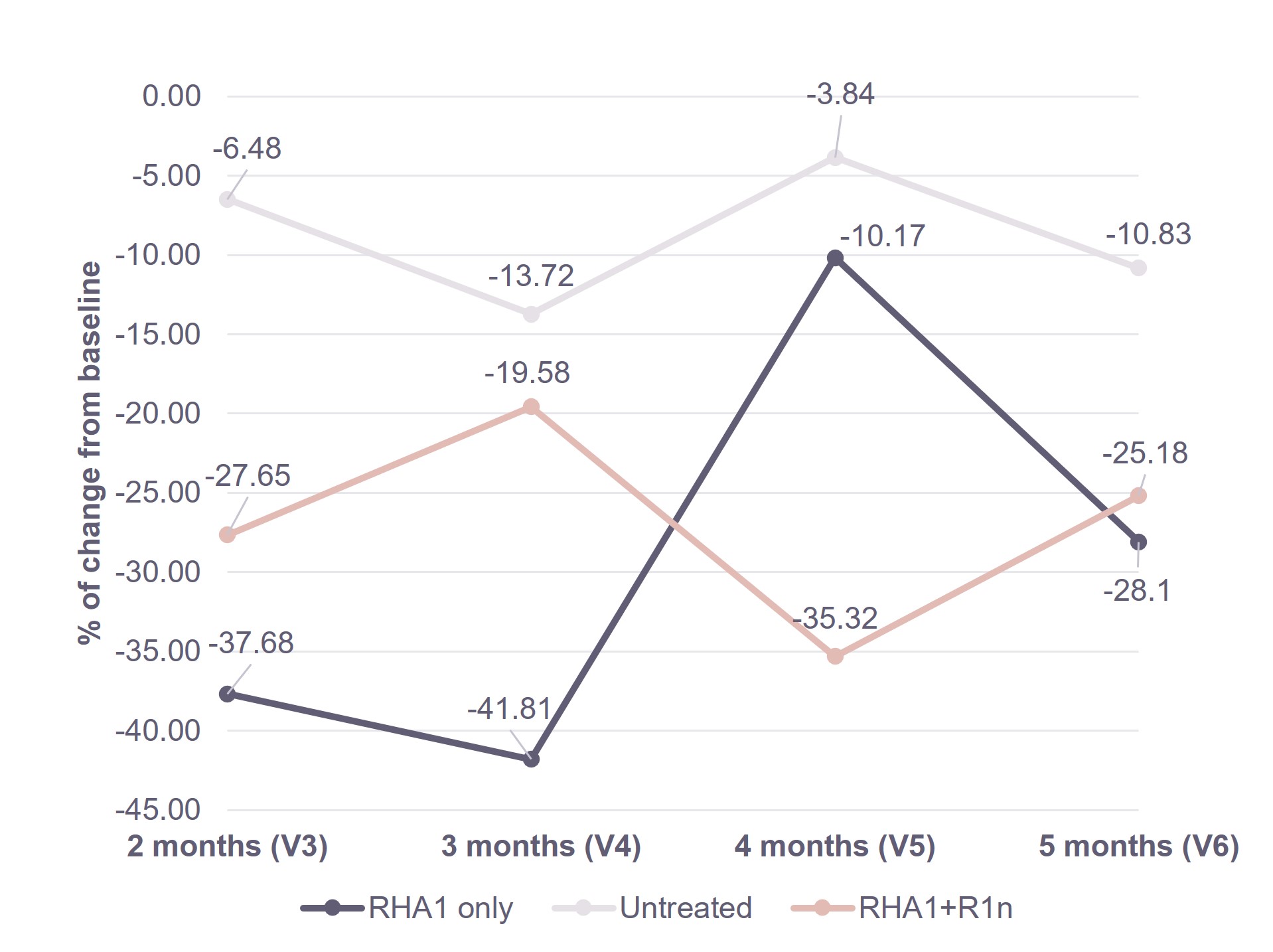

Supplement: sjaf111_Supplementary_Data [file sjaf111_supplementary_data.zip › Supplemental Figure 4B.jpg]

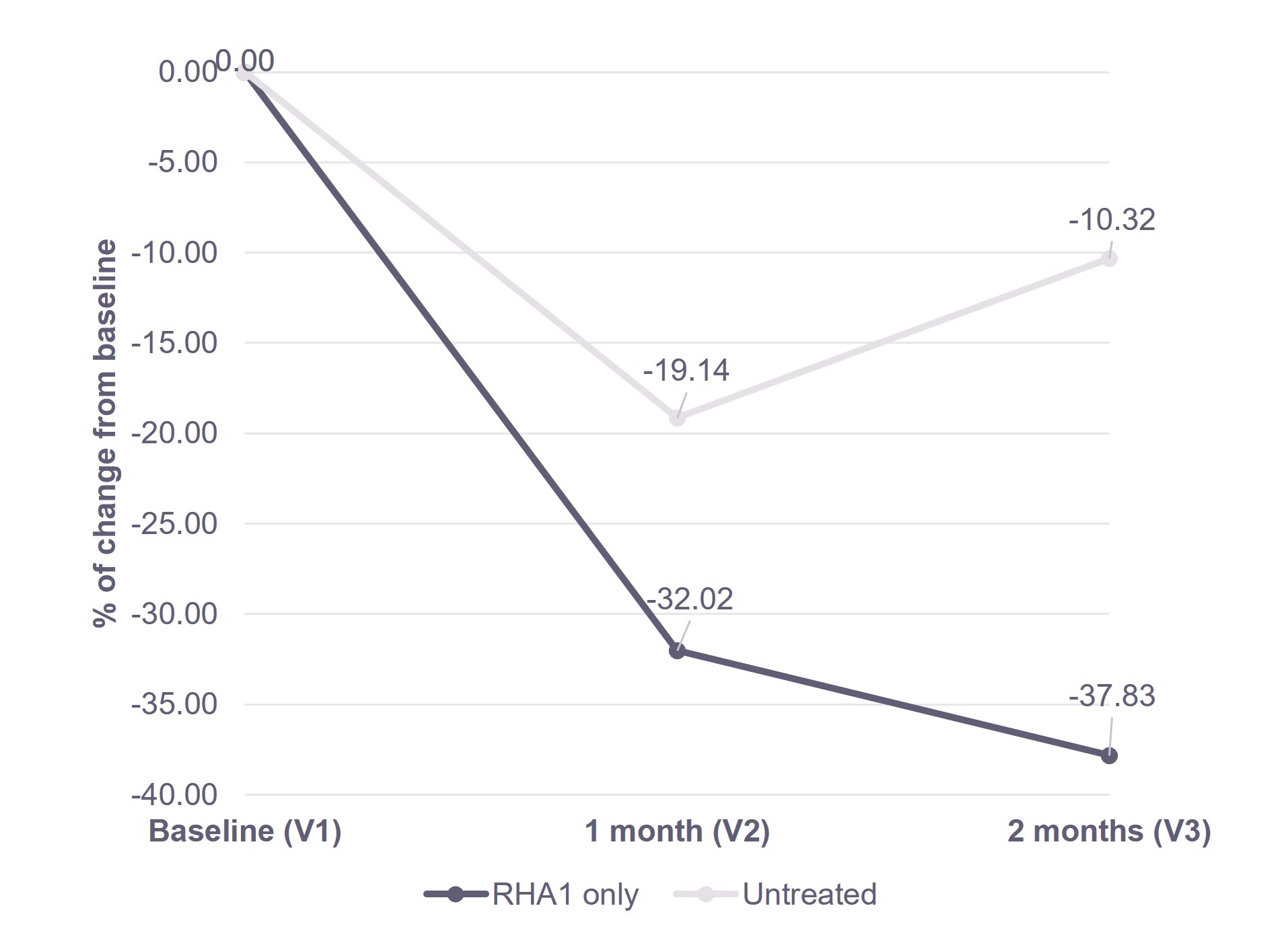

Supplement: sjaf111_Supplementary_Data [file sjaf111_supplementary_data.zip › Supplemental Figure 5A.jpg]

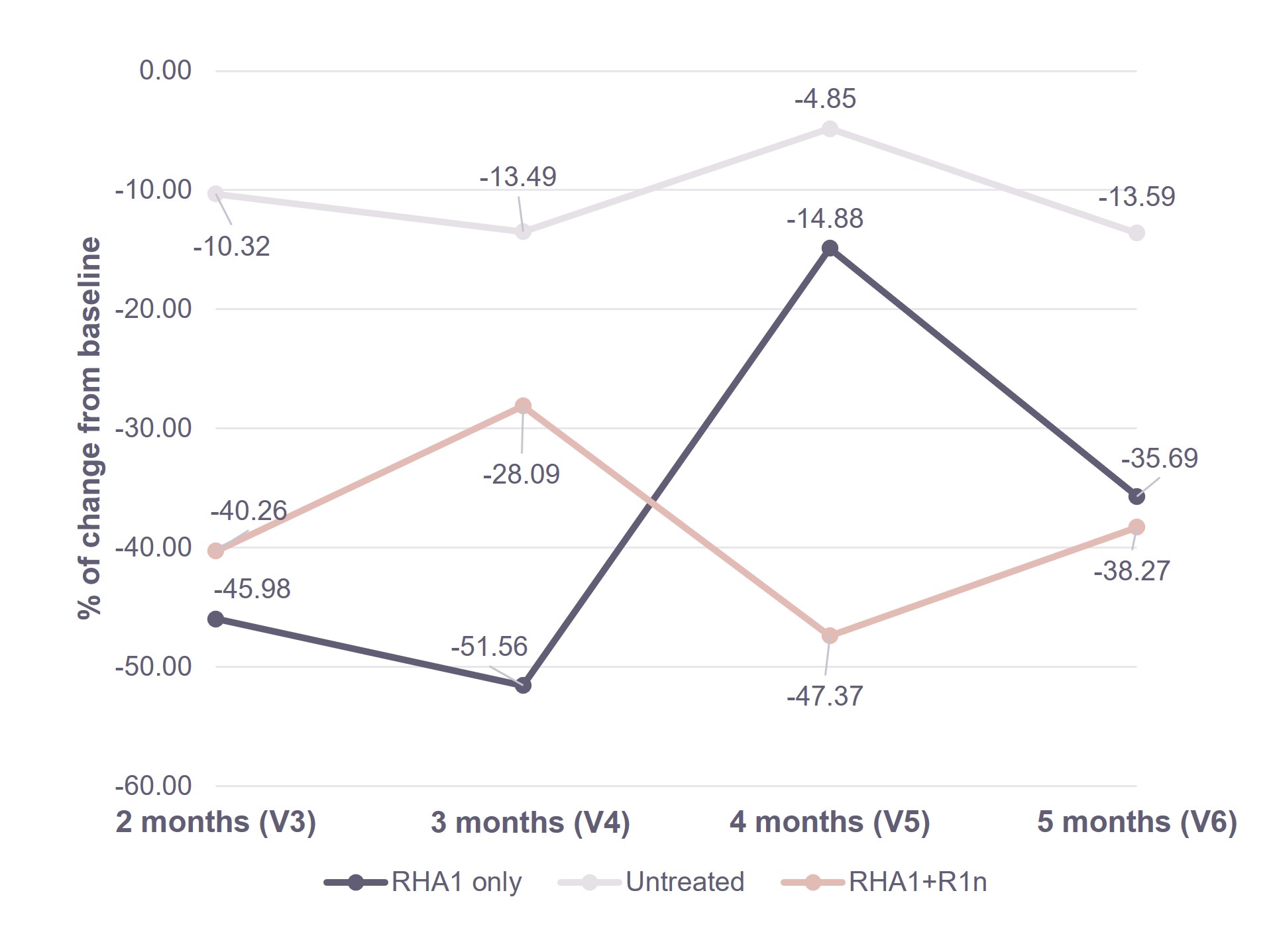

Supplement: sjaf111_Supplementary_Data [file sjaf111_supplementary_data.zip › Supplemental Figure 5B.jpg]

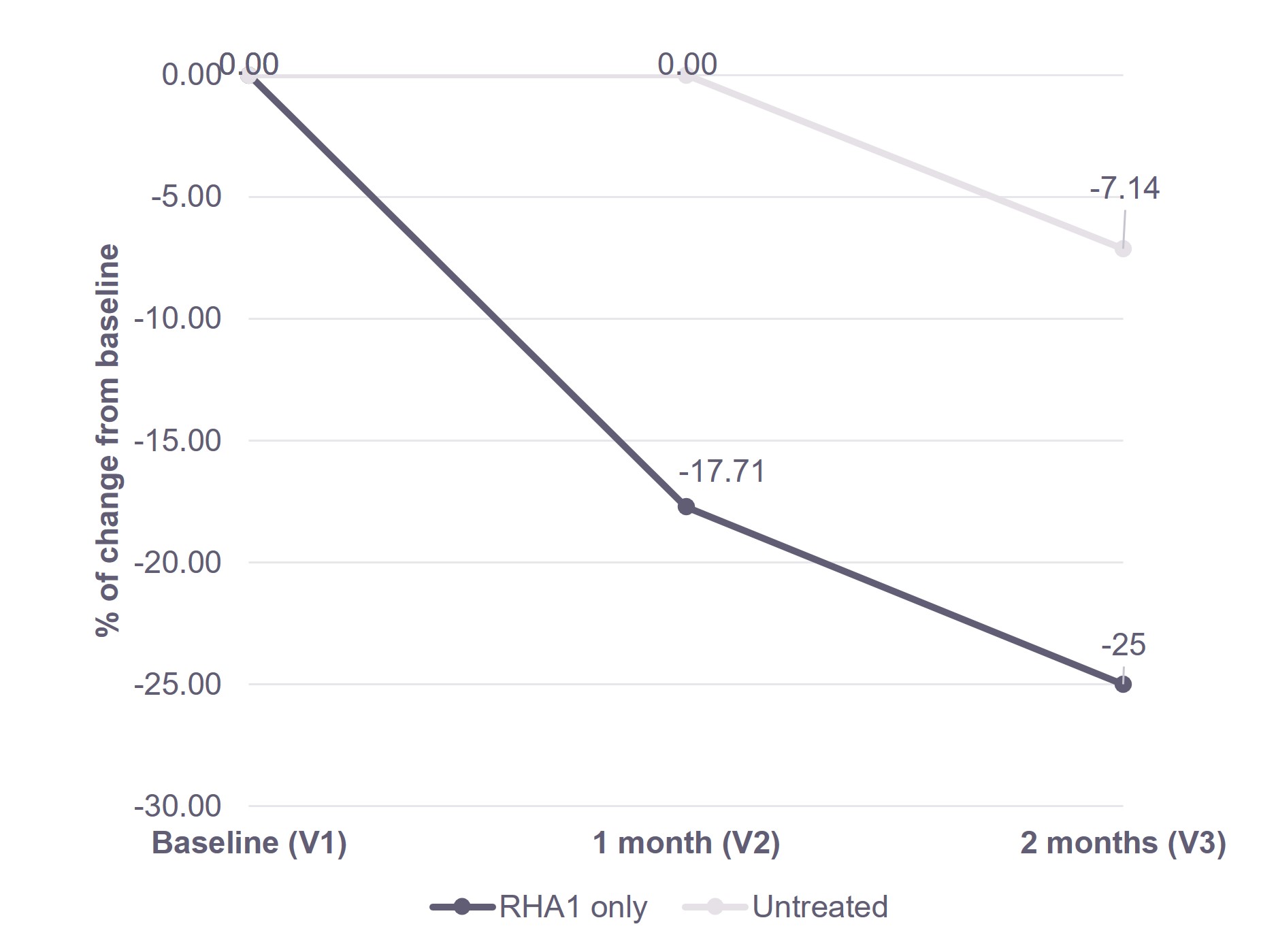

Supplement: sjaf111_Supplementary_Data [file sjaf111_supplementary_data.zip › Supplemental Figure 6A.jpg]

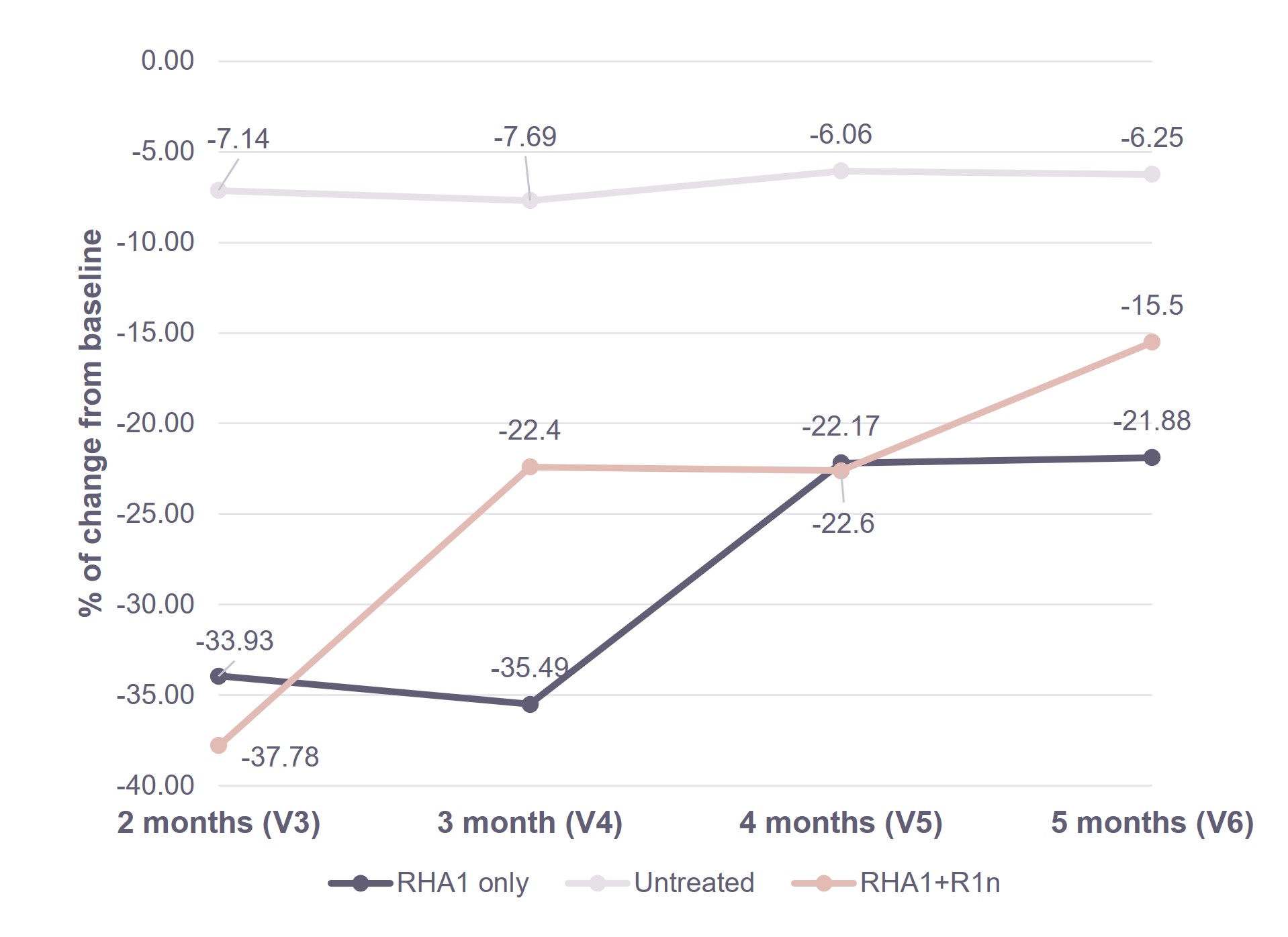

Supplement: sjaf111_Supplementary_Data [file sjaf111_supplementary_data.zip › Supplemental Figure 6B.jpg]

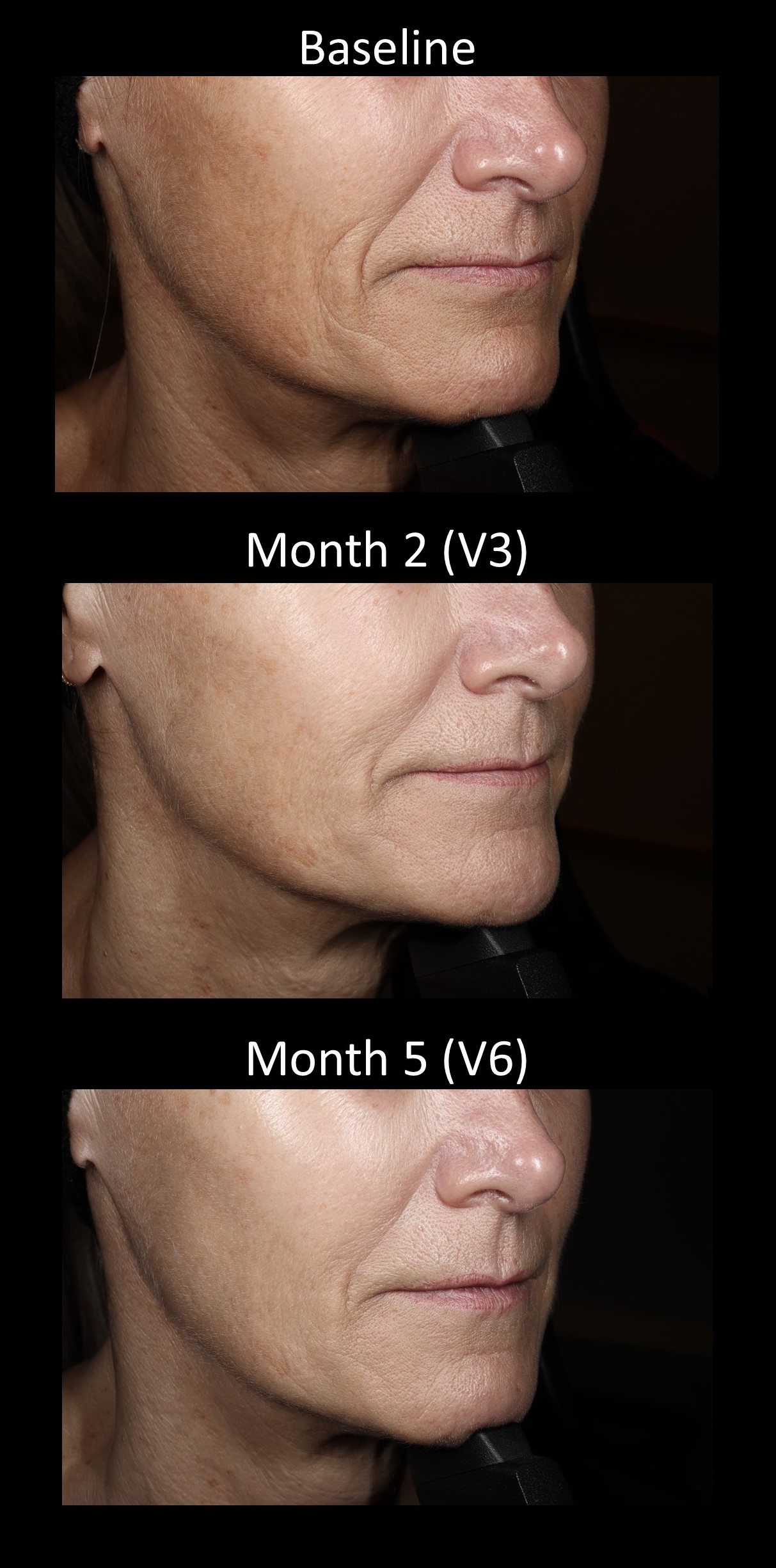

Supplement: sjaf111_Supplementary_Data [file sjaf111_supplementary_data.zip › Supplemental Figure 7A.JPG]

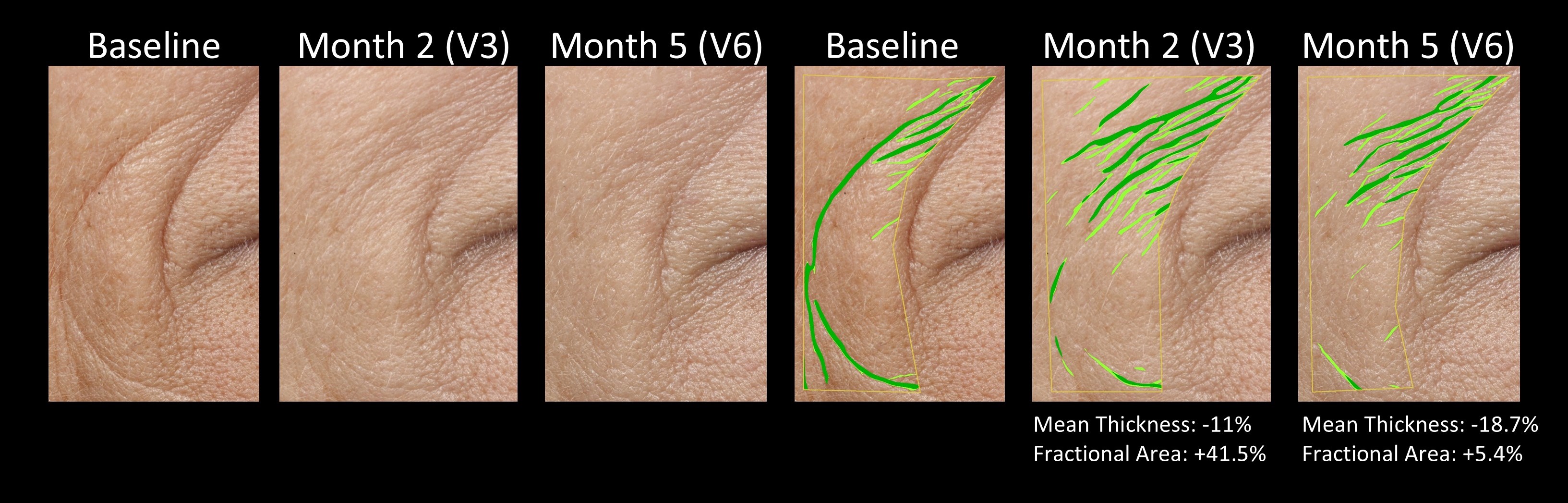

Supplement: sjaf111_Supplementary_Data [file sjaf111_supplementary_data.zip › Supplemental Figure 7B.JPG]

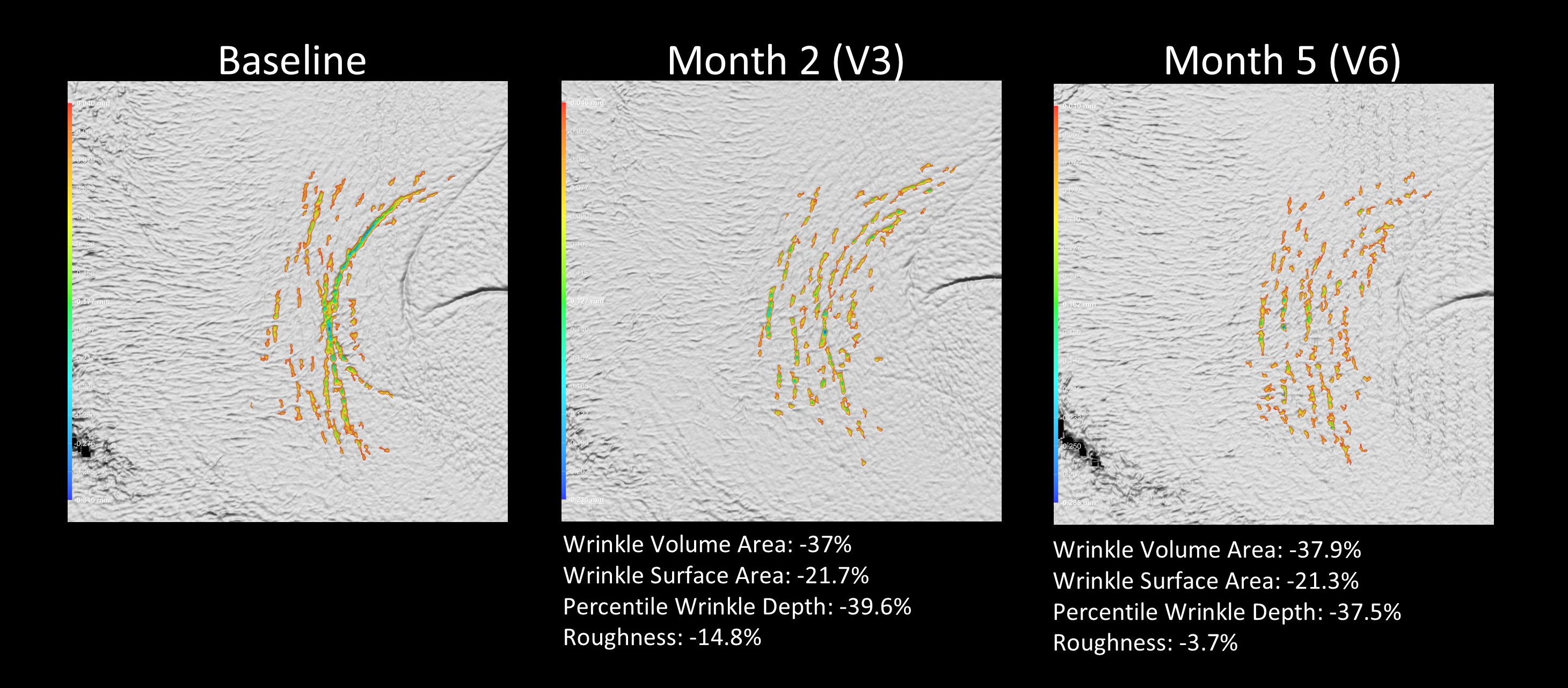

Supplement: sjaf111_Supplementary_Data [file sjaf111_supplementary_data.zip › Supplemental Figure 7C.JPG]
